# High‐Precision, Low‐Threshold Neuromodulation With Ultraflexible Electrode Arrays for Brain‐to‐Brain Interfaces

**DOI:** 10.1002/EXP.70040

**Published:** 2025-04-17

**Authors:** Yifei Ye, Ye Tian, Haifeng Liu, Jiaxuan Liu, Cunkai Zhou, Chengjian Xu, Ting Zhou, Yanyan Nie, Yu Wu, Lunming Qin, Zhitao Zhou, Xiaoling Wei, Jianlong Zhao, Zhenyu Wang, Meng Li, Tiger H. Tao, Liuyang Sun

**Affiliations:** ^1^ 2020 X‐Lab Shanghai Institute of Microsystem and Information Technology Chinese Academy of Sciences Shanghai China; ^2^ School of Graduate Study University of Chinese Academy of Sciences Beijing China; ^3^ Intelligent Communication Lab Shanghai Advanced Research Institute Chinese Academy of Sciences Shanghai China; ^4^ State Key Laboratory of Transducer Technology Shanghai Institute of Microsystem and Information Technology Chinese Academy of Sciences Shanghai China; ^5^ School of Microelectronics Shanghai University Shanghai China; ^6^ Shanghai Laboratory Animal Research Center Shanghai China; ^7^ Department of Electrical and Computer Engineering The Ohio State University Columbus Ohio USA; ^8^ College of Electronics and Information Engineering Shanghai University of Electric Power Shanghai China; ^9^ Center of Materials Science and Optoelectronics Engineering University of Chinese Academy of Sciences Beijing China; ^10^ School of Physical Science and Technology ShanghaiTech University Shanghai China; ^11^ Center for Excellence in Brain Science and Intelligence Technology Chinese Academy of Sciences Shanghai China; ^12^ Neuroxess Co., Ltd. (Jiangxi) Nanchang Jiangxi China; ^13^ Guangdong Institute of Intelligence Science and Technology Hengqin Zhuhai Guangdong China; ^14^ Tianqiao and Chrissy Chen Institute for Translational Research Shanghai China

**Keywords:** brain computer interface, brain‐to‐brain interface, flexible electrode array, neural interface, neuromodulation

## Abstract

Neuromodulation is crucial for advancing neuroscience and treating neurological disorders. However, traditional methods using rigid electrodes have been limited by large stimulating currents, low precision, and the risk of tissue damage. In this work, we developed a biocompatible ultraflexible electrode array that allows for both neural recording of spike firings and low‐threshold, high‐precision stimulation for neuromodulation. Specifically, mouse turning behavior can be effectively induced with approximately five microamperes of stimulating current, which is significantly lower than that required by conventional rigid electrodes. The array's densely packed microelectrodes enable highly selective stimulation, allowing precise targeting of specific brain areas critical for turning behavior. This low‐current, targeted stimulation approach helps maintain the health of both neurons and electrodes, as evidenced by stable neural recordings after extended stimulations. Systematic validations have confirmed the durability and biocompatibility of the electrodes. Moreover, we extended the flexible electrode array to a brain‐to‐brain interface system that allows human brain signals to directly control mouse behavior. Using advanced decoding methods, a single individual can issue eight commands to simultaneously control the behaviors of two mice. This study underscores the effectiveness of the flexible electrode array in neuromodulation, opening new avenues for interspecies communication and potential neuromodulation applications.

## Graphical abstract

Traditional neuromodulation using rigid electrodes has been limited by low precision, large stimulating currents, and the risk of tissue damage. In this work, we developed a biocompatible flexible electrode array that allows for both neural recording of spike firings and high‐precision, low‐threshold stimulation for neuromodulation. Combined with advanced decoding methods, we created a brain‐to‐brain interface that enables control of mouse behavior using human brain signals.

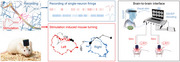

## 1 Introduction

Understanding information transfer and processing within the nervous system is a crucial challenge in neuroscience and medical research [1]. While the passive recording of neural activity yields basic neural network insights, neuromodulation enables targeted manipulation of these networks to link neural activity with specific behaviors, essential for advancing neuroscience and treating nervous system disorders [2]. Generally, neuromodulation can be achieved through various methods, including electrical, optical, magnetic, acoustic, and chemical stimulation of neural tissue to influence the surrounding neural activity [1, 3]. While various neuromodulation techniques exist, non‐invasive methods such as transcranial magnetic stimulation (TMS) are commonly used in clinical settings but are constrained by their coil geometry and centimeter spatial resolution [4]. Optical stimulation, involving genetically modifying neurons to be light‐sensitive, offers high spatial resolution but faces challenges in clinical application due to transfection [5]. In contrast, electrical stimulation stands out for its effectiveness. This technique, which stimulates neural tissue through current passed via a microelectrode [6], has not only provided profound insights into cognitive functions of specific neural circuits but also afford significant clinical applications [7]. For instance, electrical stimulation is approved for the treatment of Parkinson's disease [8] and neuropathic pain [9] and it is also actively explored as a promising therapy for major depression [10]. Furthermore, for patients who restore motor function by robotic arm, electrical stimulation was applied to evoke tactile sensations to improve robotic arm control [11].

So far, electrical stimulation for neuromodulation has mostly relied on rigid electrodes [12]. However, the mechanical mismatch between rigid electrodes and soft brain tissue often leads to the failure of effective neuromodulation. This mismatch would cause tissue damage, characterized by neuronal death and glial formation around the implant site [13]. The resultant increase in glial formation necessitates the use of higher currents for effective stimulation [13b]. This, however, expands the affected brain region beyond the intended area, leading to unintended neuromodulation. Additionally, the misplacement of the rigid electrodes during natural brain micromotions also leads to the failure of effective neuromodulation [14]. In contrast, flexible electrodes, with mechanical properties more akin to brain tissue, have shown promise in providing a more stable and safer interface for long‐term neural recording than the rigid probes [15]. Despite these advantages, research on neuromodulation via flexible electrodes is still in its early stages. Recent studies on electrical stimulation using flexible probes focused on correlating localized stimulation with neural firings, often identified through optical imaging, with initial attempts targeting simple behavioral responses [16]. However, systematic studies on inducing complex behavioral responses using flexible electrodes are still lacking. This gap in research highlights the need for comprehensive studies at the behavioral level to fully understand the potential of flexible electrodes in neuromodulation.

In this study, we have developed a flexible neural interface enabling precise turning behavior in mice. This interface, featuring an ultraflexible electrode array, ensures stable and close electrical contact with neural tissue. It allows for effective electrical stimulation with a low‐threshold current, high spatial resolution, stability, and biocompatibility. Notably, the turning behavior in mice can be precisely induced using current pulses of around 5 µA, significantly lower (by 1–2 orders of magnitude) than those required with conventional rigid electrodes [17]. Additionally, the strategic spatial distribution of the electrode array has enabled the identification of specific brain regions responsible for turning behavior, as demonstrated by distinct behavioral responses to stimulation in different regions. The durability of these flexible electrodes has been systematically validated through in vitro electrical stimulation fatigue tests and in vivo neural signal recording. The consistency of neuron firings before and after stimulation suggests the health and integrity of both electrodes and neurons post‐stimulation. To demonstrate the robustness of this flexible neural interface in controlling turning behavior, we established a brain‐to‐brain interface, allowing the transfer of human brain signals to mouse behavior. Advanced deep neural network techniques were employed to decode human brain signals, enabling simultaneous control of multiple mice in accordance with human intentions. Overall, the flexible neural interface provides an effective, stable, and safe platform for electrical stimulation in neuromodulation research and interspecies brain communication.

## 2 Results and Discussion

### 2.1 Flexible Neural Interface Enabling Both Neural Recording and Neuromodulation

To validate neuromodulation via electrical stimulation using flexible electrodes, we targeted the secondary motor cortex (M2), a brain region well‐documented in neuromodulation research [18]. Prior studies have demonstrated that the stimulation of M2 evokes general locomotion in mice [19]. Additionally, sufficient stimulation in M2 can trigger a broad combination of eye, eyelid, vibrissa, head, and limb movements [20], characterized as orienting or turning behaviors [21]. These easily observable behaviors thus can serve as indicators of neuromodulation efficacy.

**FIGURE 1:**
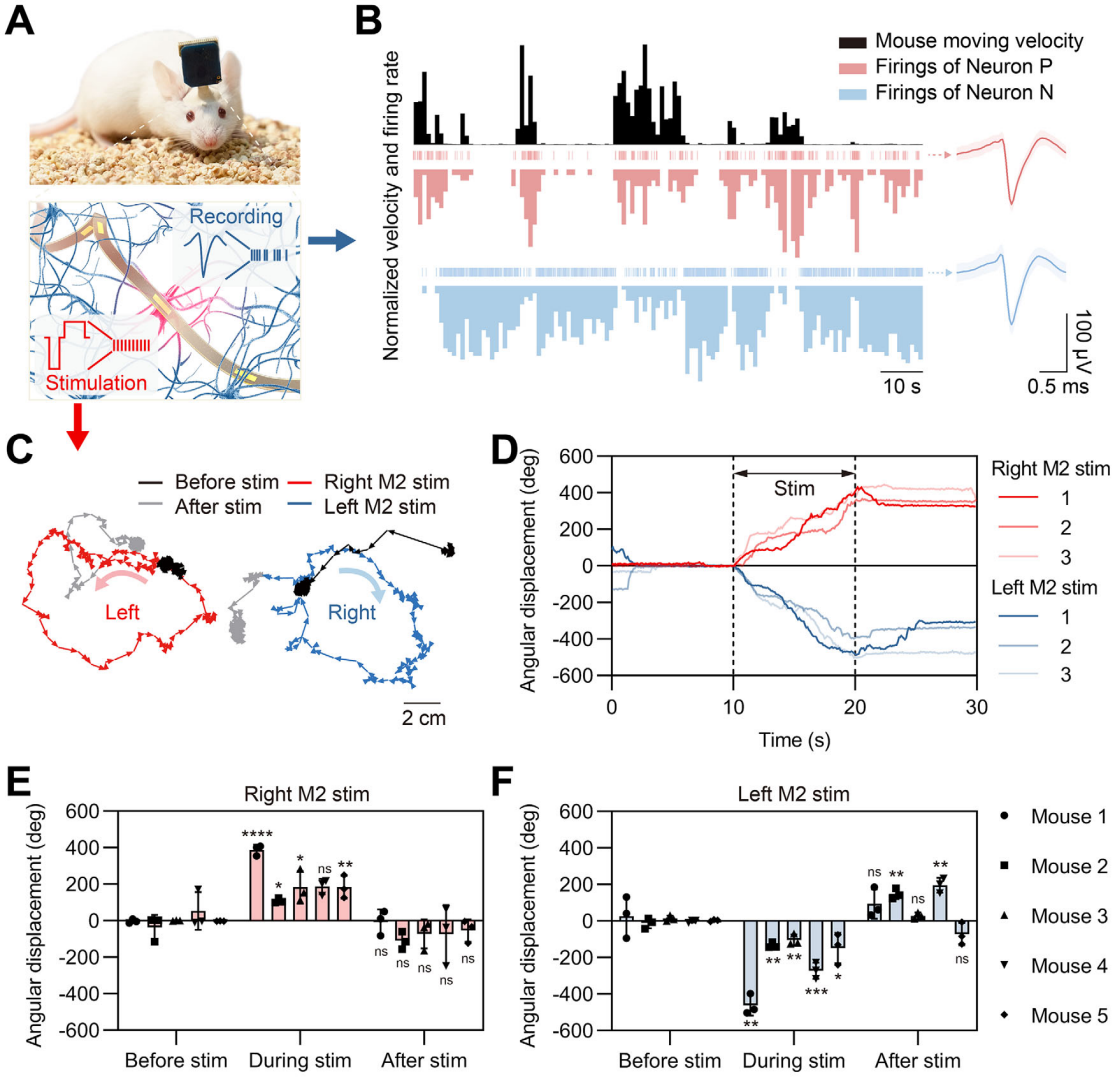
Flexible neural interface enabling both neural recording and neuromodulation. (A) Schematic diagram of the flexible neural interface for both recording and stimulation. (B) Normalized velocity of a freely moving mouse alongside simultaneous neuronal firings in the M2 region. (C) Movement trajectories of Mouse 1 before, during, and after stimulation of the right or left M2. (D) Angular displacements of Mouse 1 before, during, and after stimulation of the right or left M2. (E) Maximum changes in angular displacements before, during, and after right M2 stimulation for Mice 1–5. (F) Maximum changes in angular displacements before, during, and after left M2 stimulation for Mice 1–5. Statistical analysis was performed using two‐tailed unpaired Student's *t*‐test: ns, *p* > 0.05; *p ≤ 0.05; **p ≤ 0.01; ***p ≤ 0.001; ****p ≤ 0.0001.

Compared to optical or magnetic methods, neuromodulation by implanted electrodes has a natural advantage to be coupled with the function of neural recording (Figure 1A). The localized neural recording by the implanted electrodes provides reliable neural information for the guidance of neuromodulation. Therefore, we implanted flexible electrodes into the M2 region of mice and simultaneously recorded the localized neural signals and their movements to determine the relationship between neural activities in M2 and mouse motion. During the experiment, the mice were connected to a stimulation/recording controller (M4200, Intan Technologies) that enabled neural recording and electrical stimulation. To minimize potential interference from the wired system, we used a commutator (MMC235, Moflon) between the mice and the controller. This allowed the wire to rotate freely on one side while remaining fixed on the other, ensuring the mice could move with minimal restriction. As shown in Figure 1B, in observing freely moving mice, we discovered that the neural activities of certain neurons correlate with mouse behavior. Notably, two representative neurons, Neuron P and Neuron N, exhibited opposite responses to mouse motion. Neuron P increased its firing rate when the mouse was actively moving at high velocities, whereas Neuron N showed decreased firings. Conversely, when the mouse remained relatively stationary, Neuron P's firing rate decreased, and Neuron N's increased. The correlation between neuron firings and mouse motion validates the accurate placement of flexible electrodes in M2. Additionally, the spikes of the two neurons, characterized by high peak‐to‐peak amplitudes (113.45 and 120.34 µV) and substantial signal‐to‐noise ratios (9.43 and 7.36), demonstrate the stability and reliability of the neural recording.

To validate effective neuromodulation using flexible electrodes, electrical stimulation was conducted in the right or left M2 regions. We utilized biphasic pulses, adhering to standard neural stimulation protocols. Typically, these pulses start with a cathodic phase at the working electrode to induce membrane depolarization and action potential generation, followed by an anodic phase of equal charge [22]. This ensures charge balance, preventing charge accumulation at the electrode‐tissue interface over repeated pulsing. Standard neural stimulation typically uses pulse widths ranging from 10 to 500 µs and repetition rates between 10 and 300 Hz [23]. Following these guidelines, we designed a biphasic pulse pattern with a leading cathodic current, followed by an anodic current at half amplitude of the cathodic current. To maintain charge balance, the stimulation waveform included a 200 µs cathodic phase, a 100 µs interphase, and a 400 µs anodic phase, at a frequency of 100 Hz.

Figure 1C illustrates the movement of Mouse 1 before, during, and after stimulation of the right or left M2. This clearly shows that M2 stimulation triggers mouse motion, with right M2 stimulation inducing leftward turning and left M2 stimulation leading to rightward turning. To quantify the motion induced by stimulation, we analyzed both the linear and angular displacements of the mice (Figures S1 and S2; Figure 1D–F). The linear displacement data (Figure S1) confirms that stimulation of either side of M2 evokes motion. For Mouse 1, linear displacements during right or left M2 stimulation were 465.4 ± 54.9 mm and 474.4 ± 44.0 mm, respectively, showing significant increases from baseline measurements of 99.2±6.6 mm and 180.3±20.0 mm. More importantly, the angular displacement analysis reveals that stimulating the right or left M2 induces turning behavior in opposite directions. We defined leftward (counterclockwise) turning as positive angular displacement and rightward (clockwise) turning as negative. Figure 1D displays the angular displacements for Mouse 1, illustrating that unilateral M2 stimulation causes contralateral turning. We conducted three repeated tests for each mouse for right or left M2 stimulation and calculated the maximum angular displacement changes across three phases (before, during, and after stimulation) for five mice. The angular displacements induced during right M2 stimulation were 387.6 ± 22.9°, 109.1 ± 10.6°, 183.8 ± 72.8°, 186.7 ± 34.8°, and 183.4 ± 50.7° from baselines of −2.6 ± 9.3°, −36.0 ± 55.7°, 0.7 ± 1.8°, 53.1 ± 84.1°, and −1.8 ± 0.3°, respectively (Figure 1E). Meanwhile, the angular displacements during left M2 stimulation were −461.4 ± 45.5°, −133.6 ± 12.1°, −104.2 ± 25.4°, −271.8 ± 36.1°, and −148.4 ± 68.1° from baselines of 26.1 ± 92.4°, −10.8 ± 23.5°, 13.7 ± 12.4°, −1.4 ± 5.2°, and 3.3 ± 4.1°, respectively (Figure 1F). Note that in some cases, the angular displacement after stimulation was greater compared to that before stimulation. This may be because the mice were relatively still before stimulation due to the initial criteria, leading to minimal displacement, whereas post‐stimulation, the induced movement caused the mice to continue moving briefly before gradually returning to a stationary state, resulting in a larger displacement level. To quantify the behavioral differences induced by electrical stimulation, we conducted two‐tailed unpaired Student's *t*‐tests comparing angular displacements during and after stimulation to those before stimulation (baseline). For both right and left M2 stimulation, most tests showed significant differences in angular displacement during stimulation compared to baseline, confirming the effectiveness of the stimulation. After stimulation, the majority of tests showed no significant differences from baseline, although a few exhibited significant variations. Across the five mice (Figure S3), significant differences were observed both during (*p* ≤ 0.0001) and after stimulation (*p* ≤ 0.05), compared to before stimulation, with larger effects seen during stimulation, demonstrating the effectiveness of electrical stimulation in controlling behavior. By stimulating the right or left M2 in mice, we were able to control the mice to turn left or right, indicating that precise turning control can be achieved through targeted stimulation.

### 2.2 Electrical Stimulation With Durability, Low‐Threshold Current, and High Spatial Resolution

**FIGURE 2:**
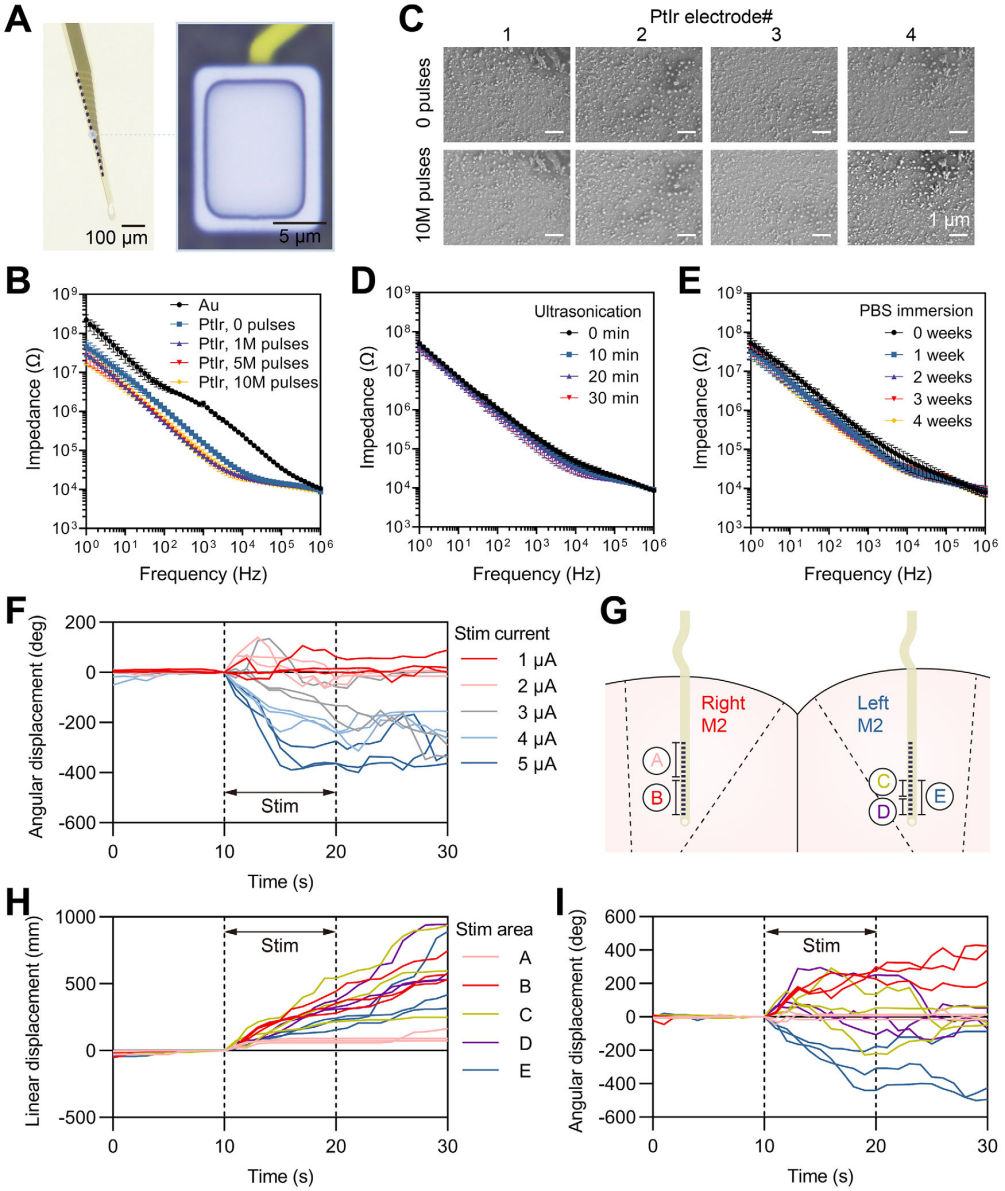
Electrical stimulation with durability, low‐threshold current, and high spatial resolution. (A) Photograph of the neural probe demonstrating flexibility in water, a close‐up of a PtIr‐coated microelectrode. (B) Electrical impedance spectroscopy results for bare Au electrodes without stimulation and PtIr‐coated electrodes before and after 1, 5, and 10 million biphasic pulse stimulations. Four Au electrodes and four PtIrcoated electrodes were tested. (C) SEM images of the four PtIr‐coated electrodes before and after 10 million biphasic pulse stimulations. (D) Electrical impedance spectroscopy results of PtIr‐coated electrodes before and after 10, 20, and 30 min ultrasonication. Ten electrodes were tested. (E) Electrical impedance spectroscopy results of PtIr‐coated electrodes before and after 1, 2, 3, and 4 weeks of PBS immersion. Sixteen electrodes were tested. (F) Angular displacements before, during, and after stimulation, with cathodic current amplitudes ranging from 1 to 5 µA. (G) Schematic diagram showing the areas of stimulation using the implanted flexible electrodes in the right or left M2 regions. (H) Linear displacements before, during, and after stimulation in brain areas A, B, C, D, and E. (I) Angular displacements before, during, and after stimulation in brain areas A, B, C, D, and E.

Flexible electrodes have demonstrated superior electrical contact with brain tissues for stable neural recording [24]. However, neural stimulation by flexible electrodes has been less studied, mainly challenged by the electrode electrochemical stability for long‐term charge injection. As the increased microelectrode impedance hampers current injection capability, it is necessary to modify the electrode with robust, low‐impedance material [13c]. To address this, we coated the bare Au electrodes with a PtIr layer as a top contact (Figure 2A). We chose PtIr rather than other commonly used conducting polymers (such as PEDOT:PSS) as the surface coating material due to its superior electrochemical stability and compatibility with standard microfabrication processes. Through PtIr coating, the electrode impedance dropped from 1.57 ± 0.23 to 0.16 ± 0.02 MΩ at 1 kHz (Figure 2B). This low impedance ensures efficient charge delivery within safe limits for both the tissue and the electrode [6], thereby enhancing the sustainability and effectiveness of electrical stimulation. To demonstrate the electrochemical stability of the PtIr‐coated electrodes, we conducted in vitro fatigue tests with repeated electrical stimulations and compared the electrical impedance spectroscopies (EIS) and corresponding scanning electron micrographs (SEM) of the electrodes before and after 10 million cycles of biphasic pulse stimulations. Using biphasic pulses with a cathodic current amplitude of 5 µA, the electrode impedance remained stable, showing no significant changes even after 10 million cycles of stimulation (Figure 2B). Additionally, SEM images revealed minimal changes in the surface morphology of the electrodes (Figure 2C). Both the EIS and SEM results confirm that the electrodes could withstand repeated electrical stimulations while maintaining their stability. To further assess the long‐term in vivo stability of the electrodes, we conducted ultrasonic tests to evaluate their mechanical robustness and PBS immersion tests to examine their stability under prolonged exposure to tissue‐like environments [25]. The ultrasonic tests, intended to simulate long‐term micromovements of brain tissue, involved subjecting the electrodes to a total of 30 min of ultrasonication. The impedance values remained stable before and after ultrasonication, indicating that the electrodes can endure mechanical stresses associated with long‐term implantation (Figure 2D). For PBS immersion, we tested electrodes at both room temperature and 60°C for 4 weeks to mimic long‐term implantation. The impedance values remained stable during the 4‐week immersion at room temperature (Figure 2E). The 60°C immersion for 4 weeks, known as an accelerated aging test, is equivalent to approximately 20 weeks at body temperature (37°C), according to ASTM guidelines [26]. Despite slight variations, the impedance remained relatively stable throughout the immersion period, indicating that the electrodes can maintain their functionality over long‐term exposure to brain tissue (Figure S4).

The threshold currents to induce effective turning behavior for mice have been determined by applying gradient rising currents. Typically, most mice require a stimulating current exceeding 3 µA to achieve effective turning. Taking one mouse for a representative example, we applied stimulating current with amplitudes varying between 1 and 5 µA to the left M2, and recorded the angular displacements of the mouse before, during, and after stimulation (Figure 2F). Within the 10 s before electrical stimulation, the mouse remains relatively stationary. However, even with the application of just 1 µA of electrical stimulation, the mouse exhibits simple behavioral responses but not orienting behavior. Under low current conditions of 1 to 3 µA, the mouse's turning behavior does not show a clear direction selectivity. However, upon increasing the current to 4 to 5 µA, the mouse demonstrates a stable rightward turning with consistent negative growth in angular displacement, registering changes of −236.9 ± 6.6° and −333.4 ± 41.9°, respectively. Compared to the tens to hundreds of microamperes used in conventional rigid electrodes, the stimulating current for flexible electrodes, in the range of a few microamperes, was significantly reduced by 1–2 orders [27], enabling stable and consistent motion modulation.

In addition, finite element simulations further emphasized the lower thresholds of flexible electrodes in comparison to the rigid electrodes (Figure S5). These simulations assessed the neuron activation capability of a flexible electrode array against a same‐sized rigid electrode array and a rigid microwire. To stimulate the same number of neurons as the flexible electrode array at a 5 µA current, the rigid electrode array required 8.9 µA, which is 1.78 times the current needed by the flexible array. Besides, the rigid microwire required 62.5 µA, equivalent to 12.5 times the current of the flexible array, to achieve similar levels of neuronal activation. Thus, both the simulations and experimental results underscore the low‐threshold stimulation offered by the proposed flexible electrode array.

Next, we investigated the spatial resolution of neuromodulation using the flexible probe. The probe features densely packed electrodes, with 16 electrodes distributed along each shank covering a total range of 450 µm, enabling high selectivity for targeting specific brain areas. As illustrated in Figure 2G, we conducted stimulation across different regions within the M2 area to assess variations in induced mouse behaviors. Initially, to determine the effective depth for controlling turning, we targeted areas A (−760 to −550 µm) and B (−1000 to −790 µm) in the right M2. The linear displacements induced by stimulation in areas A and B were 73.1 ± 12.8 and 359.6 ± 67.0 mm, respectively, compared to baselines of 12.3 ± 12.5 and 28.2 ± 17.2 mm (Figure 2H and Figure S6). This indicates only a slight motion evocation by stimulating area A, while stimulating area B showed a significant activation of motion. The angular displacements revealed that stimulation of the deeper right M2 (area B) effectively induced leftward turning (268.1 ± 29.3°), whereas stimulation of the shallower right M2 (area A) elicited only minor, initial rightward turning (−28.2 ± 15.3°), which then returned to near‐baseline (1.5 ± 13.3°) (Figure 2I). These results suggest that deeper regions of M2 are pivotal for controlling turning behavior. Further focusing on the deep regions, we stimulated deep left M2 (area E: −1000 to −790 µm) and two narrower subregions within it (area C: −880 to −790 µm; area D: −1000 to −910 µm). Both angular and linear displacements confirmed that stimulation in these areas significantly influenced motion. The induced linear displacements of subregions C and D, and the entire deep area E were 367.3 ± 133.5, 336.9 ± 28.4, and 209.8 ± 38.3 mm, respectively, from baselines of 14.8 ± 3.4, 3.4 ± 4.8, and 30.0 ± 21.2 mm. However, targeting the subregions did not result in selective turning; in contrast, stimulation of the entire deep area consistently led to stable rightward turning (−307.7 ± 108.0°). These findings suggest that narrow stimulation is insufficient for inducing directional turning, while broader stimulation reliably triggers specific unidirectional movements, likely due to the involvement of multiple neurons within a relatively broad area.

### 2.3 Bio‐Compatibility of Flexible Neural Interface for Electrical Stimulation

A stable neural interface requires not only the robustness of the implanted electrodes but also the health and integrity of the surrounding tissue. Having established the electrode durability to repeated electrical stimulation (Figure 2B), we now turn our focus to its biocompatibility. We thoroughly investigate the flexible neural interface's interaction with neural tissue, encompassing both the monitoring of neural activity and the assessment of tissue integrity after extended periods of stimulation and implantation.

**FIGURE 3:**
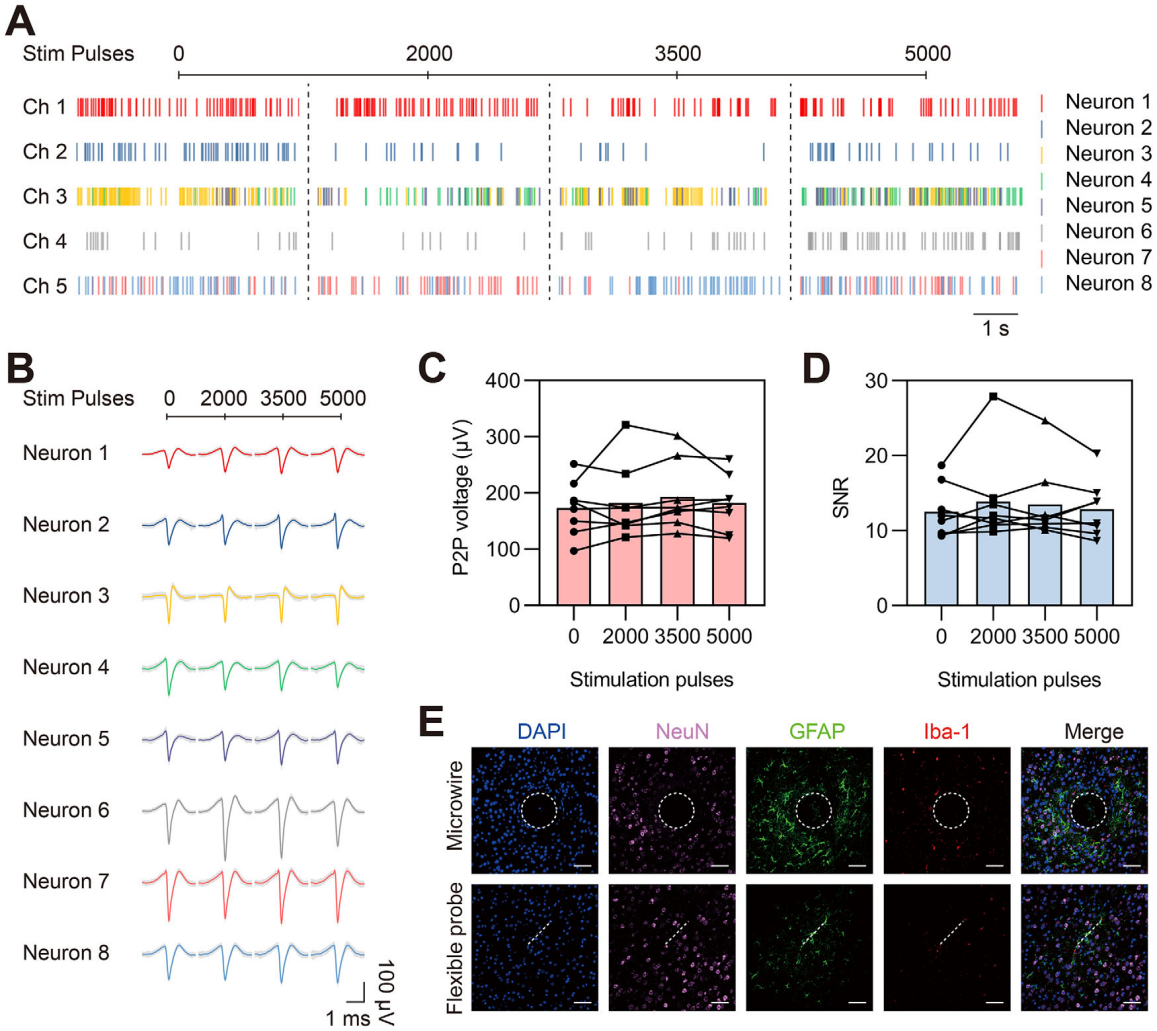
Bio‐compatibility of flexible neural interface for electrical stimulation. (A) Firing patterns of eight neurons (Neuron 1–8) recorded by five electrodes (Ch1‐5) before and after receiving 2000, 3500, and 5000 stimulation pulses. (B) Action potential waveforms of the eight neurons before and after stimulation with 2000, 3500, and 5000 pulses. (C) Peak‐to‐peak voltage measurements of action potentials from the eight neurons before and after 2000, 3500, and 5000 pulses of stimulation. (D) Signal‐to‐noise ratios of neural firings from the eight neurons before and after stimulation with 2000, 3500, and 5000 pulses. (E) Confocal fluorescence images showing nuclei (DAPI, blue), neurons (NeuN, pink), astrocytes (GFAP, green), and microglia (Iba‐1, red) post 6‐month implantation of a microwire (diameter: 100 µm) and a flexible probe (width: 100 µm, thickness: 5 µm). Scale bar: 50 µm.

A primary measure of the interface's stability is the consistency of neural signals recorded by the electrodes before and after stimulation. Stable recording of neural activity necessitates robust electrodes, as well as the preservation of neuron functionality. As illustrated in Figure 3A, the raster plot displays the firing patterns of eight example neurons, each represented by a unique color. Notably, these neurons were consistently detected by five nearby electrodes, maintaining their activity both before and after stimulation of 2000 to 5000 pulses. The variation in firing rates did not compromise their detectability. Furthermore, the average action potential waveforms of each neuron, depicted in Figure 3B, remained consistent in shape before and after receiving 2000, 3500, and 5000 pulses. Quantitatively, both the peak‐to‐peak (P2P) voltage of the action potentials (Figure 3C) and the signal‐to‐noise ratio (SNR) of the detected neuron firings (Figure 3D) remained stable throughout the experiments. Specifically, the average P2P voltage measurements were 173.3 ± 45.3, 182.2 ± 61.4, 192.9 ± 56.0, and 181.9 ± 45.0 µV, and the average SNR values were 12.5 ± 3.3, 13.9 ± 5.5, 13.5 ± 4.6, and 12.9 ± 3.5 before and after receiving 2000, 3500, and 5000 pulses, respectively. These consistent measurements underscore the stability of both the stimulated neurons and the electrodes.

For a long‐term perspective on safety, we compared the tissue damage caused by a traditional rigid microwire (approximately 100 µm diameter) and our flexible neural probe (about 5 µm in total thickness) following a 6‐month implantation in the mouse brain. The results, illustrated in Figure 3E, show a marked difference. The rigid microwire left a roughly 100 µm hole at the implant site, causing neuron loss and the formation of a glial scar surrounding the cavity. In contrast, the flexible probe caused minimal tissue damage and neuron loss, with only a negligible amount of scar tissue observed around the implantation area. Considering the consistent neural recordings before and after stimulation, coupled with the minimal tissue damage observed after long‐term implantation, it is evident that the flexible neural electrodes offer a safe and reliable method for electrical stimulation, ensuring sustained in vivo safety and functionality.

### 2.4 Brain‐to‐Brain Interface for Controlling Mouse Turning via Human Brain Signals

The brain‐to‐brain interface (B2BI) represents a pioneering technology that establishes a direct communication pathway between individual brains, providing a novel interaction method beyond traditional communication [28]. Following our comprehensive demonstration of robust neuromodulation using flexible electrodes, we applied this technology to a B2BI system that combines neuromodulation with human brain signal detection and decoding. This interface enables the direct control of mouse movements using the decoded human brain signals.

**FIGURE 4:**
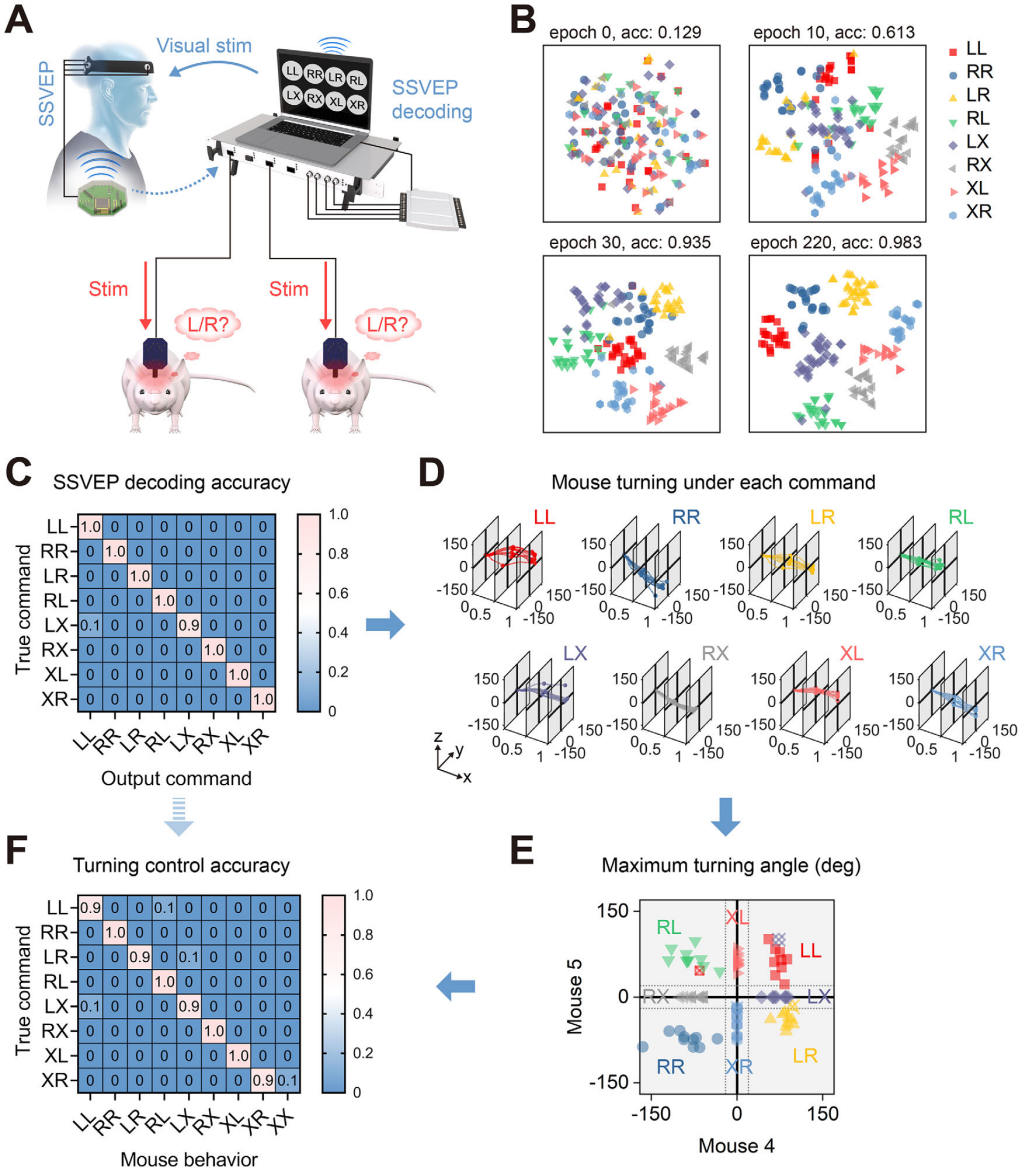
Brain‐to‐brain interface for controlling mouse turning via human brain signals. (A) Schematic illustration of the brain‐to‐brain interface designed to control mouse turning based on human brain signals. (B) The *t*‐distributed stochastic neighbor embedding (*t*‐SNE) visualization with epochs varying from 0 to 220. (C) Confusion matrix of SSVEP decoding accuracy for controlling Mice 4 and 5. (D) Angular displacements of Mice 4 and 5 during 1‐s stimulations for each command, where the *x*‐axis represents stimulation time (s), *y*‐axis represents angular displacements (degree) of Mouse 4, and *z*‐axis represents angular displacements (degree) of Mouse 5. (E) Maximum turning angles achieved by Mice 4 and 5 in response to commands. (F) Confusion matrix of turning control accuracy of B2BI system.

As depicted in Figure 4A, the proposed B2BI system connects one human and two mice, with the laptop wirelessly linked to the EEG acquisition system and wired to the electrical stimulation controller for the mice, allowing the human subject to issue eight different commands that control both mice simultaneously. The laptop independently manages data acquisition, real‐time processing, and analysis, while the DNN model, trained on a separate server, is executed in real‐time on the laptop to generate the necessary commands. These commands are LL, RR, LR, RL, LX, RX, XL, and XR. Each pair of letters represents specific motion commands for the two mice: the first letter corresponds to the command for the first mouse and the second letter for the second mouse. L denotes a left turn by stimulating the right M2, R denotes a right turn by stimulating the left M2, and X indicates no stimulation. For human brain signal detection, we utilized the steady‐state visual evoked potential (SSVEP) paradigm [29], known for its superior signal‐to‐noise ratio and clear features in both temporal and frequency domains, which enhance classification accuracy and transfer rates [30]. In each trial, SSVEPs are collected from the human occipital region when participants focus on the visually stimulating flickering patterns. Each trial included a 2‐s cue period for the target indication, followed by a 1‐s stimulation period with specific flickering frequencies to evoke the SSVEPs. After the 1‐s stimulation, the B2BI system rapidly processes the data and generates corresponding commands within 1.5 ms, initiating a 1‐s continuous electrical stimulation for the mice. The frequencies of the eight targets ranged from 8 to 15 Hz, with a 1 Hz interval between each, and each frequency was accompanied by phase offsets of 0, π/2, π, 3π/2, 0, π/2, π, 3π/2, respectively. For instance, typical SSVEP data in response to a 10 Hz target was analyzed in both the time and frequency domains (Figure S7). The time‐domain data displays distinct SSVEP features, while the frequency‐domain data clearly shows the 10 Hz fundamental frequency and its harmonics, confirming the validity of the signal. A specifically tailored deep neural network is employed to decode elicited brain signals into predefined commands by integrating inputs from both time and frequency domains. Its streamlined and efficient design (Figure S8), coupled with parallel computations on a GPU, ensures swift processing times not exceeding 1.5 ms.

To evaluate the general performance of our neural network in signal decoding, we first trained the model using data from four different subjects and assessed its accuracy with data from an additional subject. Remarkably, as shown in Figure S9, the inter‐subject decoding accuracy achieved 92.5%, demonstrating the model's robust generalizability and performance. To further enhance decoding accuracy for optimal integration with the B2BI system, we performed experiments in single‐subject inter‐session scenarios, training and evaluating exclusively with data from one participant. We systematically optimized the model's decoding performance by adjusting signal lengths, the number of repeating blocks, and the number of channels, as detailed in Figure S10A–C. Notably, we identified optimal parameters (1‐s signal length, ten training trials, and eight channels) that yielded an impressive average 8‐target identification accuracy of 98.1%, as shown in the averaged confusion matrix (Figure S10D). These results highlight the deep neural network's capability to accurately decode SSVEP signals, thereby enabling precise command execution within the B2BI system.

To enhance understanding of the neural network's classification mechanisms, we extracted the encoded features before the final fully connected layer and employed *t*‐distributed stochastic neighbor embedding (t‐SNE) to visualize the distribution of these features (Figure S9A and Figure 4B). For the single‐subject inter‐session decoding (Figure 4B), it is evident that as training progressed, the encoded features became increasingly distinct and clustered more discernibly. During the early training process, from epoch 0 to 220, model accuracy improved significantly from 0.129 to 0.983. Similarly, the model accuracy of the inter‐subject decoding reached 0.9 with epoch 70 (Figure S9A). This pattern of clustering reveals that the network has effectively learned to map input signals into a higher‐dimensional space, in which the distribution of the data can achieve tighter clustering for features within the same class and greater separation between different classes.

Integrating human brain signal decoding with mouse brain stimulation, we developed a brain‐to‐brain interface system that enables direct control of mouse behavior by human brain signals. Specifically, two sets of mice, Mice 4–5 and Mice 2–3, were manipulated by one human participant through this interface. Each set of mice and the human participant underwent 80 tests, with eight commands repeated ten times across eight blocks. Short intervals of 2 s were placed between repetitions within each block. To prevent fatigue in both the human participant and the mice, a break of several minutes was taken after each block, and the next block was initiated when the participant observed that the mouse was relatively stationary and ready for the next stimulation session. The detailed step‐by‐step results of the B2BI system for controlling Mice 4–5 are illustrated in Figure 4C–F. Figure 4C demonstrates the human SSVEP decoding accuracy for the first step, showing that most output commands decoded from human SSVEPs closely matched the preset true commands, achieving a total accuracy of 98.75%. Upon receiving these commands, Mice 4 and 5 responded accordingly. The induced turning angles under each command are detailed in Figure 4D, with the *x*‐axis representing the entire 1‐s duration of the command, the *y*‐axis showing the turning angle of Mouse 4, and the *z*‐axis depicting the turning angle of Mouse 5. Positive angles indicate left turns, while negative angles indicate right turns, clearly showing that the behaviors of the mice varied significantly under different commands. The three‐dimensional figures of mouse turning angles across the 1‐s command period provide a comprehensive view of the changes. For enhanced visualization, we transformed these three‐dimensional images into two‐dimensional images by extracting the maximum turning angles of each trial and plotting them on a *yz*‐plane to directly reflect the induced turning of these two mice under each command. A valid turn was defined as one where the absolute value of the turning angle exceeded 20°. Solid dots denote correct responses matching the true commands, while hollow dots with forks indicate incorrect behavior. Results presented in Figure 4E indicate that most dots were accurately positioned in the predicted regions on the *yz*‐plane, demonstrating that the control of mouse turning largely aligned with the given commands. Furthermore, we illustrated the confusion matrix to correlate mouse behavior with the true commands (Figure 4F). We noted a behavior pattern labeled “XX,” indicating that both mice did not exhibit an effective turning response, a scenario not included in the preset commands as it corresponds to no stimulation. As calculated from the confusion matrix, the overall accuracy of turning control for Mice 4–5 by human signals was 95%. Similarly, the control results for Mice 2–3, as shown in Figure S11, demonstrated a total control accuracy of 97.5%. These findings highlight that, by combining flexible electrodes for robust neuromodulation with advanced decoding techniques, the B2BI system facilitates precise communication from one human brain to multiple animal brains, marking a significant step forward in the development of a scalable 1:*N* brain‐to‐brain interface.

## 3 Conclusion

In this study, we developed a flexible neural interface that allows for precise motion control in mice using flexible electrodes for brain stimulation. This approach represents a significant improvement over traditional rigid electrodes by showing that minimal current stimulations around 5 µA are adequate to induce effective behavioral responses. The integration of a flexible electrode array enables precise, programmable neuromodulation, demonstrated by distinct behavioral responses when various brain regions are stimulated at high spatial resolution. Furthermore, the low‐threshold, localized stimulation provides a safe option for neuromodulation, evidenced by stable neuron firing patterns before and after stimulation, as well as reduced neuron loss and glial formation after 6 months, compared to traditional rigid microwires. Expanding on the capabilities of the flexible neural interface, we adapted this technology to a brain‐to‐brain interface, establishing a direct communication pathway from the human brain to the mouse brain. Using advanced deep neural networks to decode human brain signals, a single individual can control two mice simultaneously with averaged accuracy exceeding 95%. These results highlight the robustness of neuromodulation using flexible electrodes, offering a safe and effective platform for motion control and potentially other neuromodulation applications.

## 4 Experimental Section

### 4.1 Animal

Adult male ICR mice (7 weeks) were used for all of the animal studies. All animal tests were approved by the Ethics Committee for Animal Management at the Shanghai Laboratory Animal Research Center (approval number: PA202300901).

### 4.2 Flexible Neural Probe Fabrication

Flexible neural probes were fabricated using standard microfabrication techniques described below (Figure S12):

- Step 1: The fabrication process began with the patterning of the sacrificial layer, utilizing a double‐resist technique to facilitate the lift‐off procedure. Initially, lift‐off resist (LOR 5b) was spin‐coated onto the wafer at 4000 rpm for 40 s, followed by soft baking at 180°C for 5 min. Subsequently, a 2 µm layer of positive photoresist (LC100A) was spin‐coated and baked at 110°C for 90 s. The photoresist layers were then patterned through photolithography and developed using tetramethylammonium hydroxide (TMAH). Following that, a Cr/Ni layer with a thickness of 5/150 nm was deposited via electron beam evaporation, and the sacrificial layer pattern was formed by lifting off residual metals using acetone and TMAH developer.
- Step 2: The next stage involved the deposition of the bottom polyimide (PI) encapsulation layer. To enhance adhesion, tackifier VM652 was initially coated and cured before applying PI precursor (PI2610) by spin coating at 3000 rpm for 45 s. The precursor layer was subsequently cured in a vacuum oven with nitrogen inlet through a stepwise temperature process, culminating in a final curing temperature of 350°C.
- Step 3: For the fabrication of the metal interconnects of the probe, a single negative photoresist (AZ nlof 2020) was employed in place of the double‐resist approach to achieve higher patterning resolution. The photoresist was spin‐coated at 3000 rpm for 30 s, soft‐baked at 110°C for 110 s, exposed for 5 s, post‐baked at 110°C for 90 s, and developed for 40 s. A Ti/Au/Ti (5/100/5 nm) layer was then deposited via evaporation and patterned using lift‐off in acetone.
- Step 4: Backend pads were fabricated using a method similar to that of the sacrificial layer, but with the metal composition adjusted to Ti/Ni/Au (5/150/50 nm).
- Step 5: The top PI encapsulation layer was formed following the same procedure as for the bottom PI layer.
- Step 6: The shape of the neural probe was defined through reactive ion etching (RIE) of the PI layer. A 100 nm Al layer was sputtered onto the wafer to serve as a hard mask, which was subsequently patterned using photolithography with positive photoresist (LC100A) and wet etched using aluminum etchant.
- Step 7: Oxygen RIE was then employed to etch the PI, thereby defining the probe profile and exposing the electrodes and backend pads. The remaining Al mask was removed using aluminum etchant.
- Step 8: The final step involved fabricating the top electrodes of the probe. This was accomplished through double‐resist photolithography and lift‐off, with a 5/150 nm layer of sputtered Ti/PtIr (80:20 wt%) used for the top electrodes to enhance electrical properties.

### 4.3 Flexible Neural Probe Assembly and Implantation

As the flexible probe was too soft to penetrate the brain tissue, the tip‐etched tungsten wires were utilized to assist probe implantation as we previously proposed [31]. First, the front of the tungsten wires was etched in NaOH solution with a positive voltage of 8 V applied until a T‐shaped tip was formed. Then, two flexible shanks with holes in the front were sleeved into the tip‐etched tungsten wires and temporally bonded by dissolvable polyethylene glycol (PEG). In operation, the shanks were implanted into the secondary motor cortex (M2: Anteroposterior (AP) +1.5 mm, mediolateral (ML) ±0.75 mm, dorsoventral (DV) −1 mm) of the left and right brain. Following implantation, the PEG was dissolved using a phosphate‐buffered saline (PBS) solution. Subsequently, the tungsten wires were carefully extracted, and the device was fixed using dental cement.

### 4.4 Finite Element Simulation

Three‐dimensional finite element (FEM) simulation models were developed based on the COMSOL Multiphysics to examine the variations in neuron activation among the flexible electrode array, the rigid electrode array, and the rigid microwire. The number of neurons that could be triggered under particular stimulation settings was investigated using these models. Brain tissue was simulated using a cube whose sides measured 3 mm. Surfaces that matched the size of the real electrodes were placed on the cube's bottom to replicate electrodes. These surfaces’ boundary conditions were established as constant current sources. Both the flexible and rigid electrode arrays were intended to have the same electrode sizes and configurations. The electrodes were modeled as eight 16 µm × 12 µm rectangular surfaces that were positioned 30 µm apart. Since conventional rigid microwires are only conductive at the tip, the rigid microwire was simulated by a 100 µm‐diameter circular surface. Given that rigid probe implantation would result in glial formation surrounding the probe, both the rigid electrode array and rigid microwire were designed to be covered with a 40 µm thick layer of glial. The brain tissue's electrical conductivity was set to 0.2 S m^−1^, and the relative permittivity was set to 88.9 [16, 32]. The glial scar's electrical conductivity was tuned to 0.166 S m^−1^, and the relative permittivity was set to 88.9 [16, 33]. As the necessary condition for neuron activation, we used the current density threshold to calculate the number of neurons that can be triggered under specific current values. Current density is a key parameter affecting neural activity. Previous research reports that the current density threshold for effective neural modulation is approximately 1000 A m^2^ [34]. Additionally, the M2 region has a neuron density of 135,801 m^−3^ [35]. Thus, the number of activatable neurons can be calculated by multiplying the neuron density of M2 region by the volume of brain tissue with a current density higher than 1000 A m^−2^ during specific electrical stimulation. We parametrically scanned from 1 to 100 µA, setting the electrode's current output as a variable parameter to examine the relationship between the current magnitude and the range of activatable neurons.

### 4.5 Spike Sorting and Analysis of Neural Signals From Mice

Spike sorting of raw neural signals from the mouse brain was performed using MountainSort. Initially, the data underwent a bandwidth filter with the frequency set to 600–6000 Hz. This step was crucial to eliminate low‐frequency components and high‐frequency noise, thereby isolating the neural signals' essential frequency band. Subsequently, the principal component analysis (PCA) method was employed to distill features from the data on each channel. These features were then clustered using the ISO‐SPLIT method, allowing for the identification of multiple neurons. This spike sorting process enabled us to extract critical information, such as the time series, waveform, signal‐to‐noise ratio, and recorded channels for each neuron's firings from the multi‐channel raw neural signal data. All subsequent analyses and processing were conducted using MATLAB.

### 4.6 Human SSVEP Signal Collection

For the collection of non‐invasive EEG signals, all participants provided informed written consent. We employed semi‐dry, pre‐gelled electrodes that are designed to optimize both user experience and signal quality. These electrodes were arranged in a headband configuration to enhance signal recording, specifically in the human occipital region. Since the wearable electrode cap posed no physical risks to participants, ethical approval was not required.

EEG data were collected at a sampling rate of 1000 SPS using an 8‐channel EEG cap. The electrodes were positioned in the occipital region according to the international 10–20 system, specifically at channels O1, POz, PO3, PO5, Oz, PO6, PO4, and O2 [36]. The reference and ground electrodes were placed at FP1 and FP2 on the forehead for comfort and ease of use. These channels were selected sequentially (from O1 to O2) to analyze decoding accuracy. A custom‐designed wireless EEG device was used to record raw signals from the human brain. This device consists of two primary functional modules: a sensing module for EEG recording and sampling and a transmission module for wirelessly transmitting the data to a computer for processing. The sensing module utilizes the ADS1299 as the analog front end to sample EEG signals from the electrode cap with a resolution of 0.298 µV. The transmission module employs the ESP12E to facilitate high‐speed Wi‐Fi communication. Before decoding, the collected EEG data were preprocessed by subjecting to notch filtering at 48–52 Hz and a second‐order Butterworth bandpass filter at 4–100 Hz to remove power line interference and other significant noise.

### 4.7 Human SSVFP Decoding Based on Deep Neural Network

The SSVEP decoding utilized an advanced deep neural network (DNN). As illustrated in Figure S8, the network is ingeniously architected with dual branches that concurrently process signals from the time domain and frequency domain. The time domain branch starts with reformatting the input signal into tensors with a configuration of (batch, 1, channels, time steps). These tensors are then transformed through a sequence of 2D convolutional layers, max pooling, and linear layers. In particular, 2D convolution operations are chosen over 1D convolution due to their ability to process both temporal and inter‐channel signals simultaneously, which enhances computational efficiency and performance. Concurrently, the frequency domain branch applies the short‐time Fourier transform to the input signal to compute the Fourier transform over short, overlapping segments. This operation yields time‐frequency maps with dimensions (batch, channels, height, and width). Residual blocks serve as sophisticated image feature extractors, decoding these time‐frequency maps. To expand the receptive field substantially, dilated convolutions with a broad kernel size of [9, 13] are employed in the network design. The processed outputs of the two branches are then concatenated before being conveyed through a fully connected layer, which furnishes a one‐hot encoded vector indicative of the classification verdict. To bolster model generalization and avoid overfitting, we have integrated batch normalization and dropout layers within the network.

The SSVEP experiments involved five human participants, with each trial recording 1‐s EEG signals. In the inter‐subject validation experiments (Figure S9), the training dataset comprised a total of 1120 trials of EEG signals collected from four participants, while the test dataset included 120 trials from an additional participant. For the single‐subject validation experiments (Figure S10), the training dataset consisted of 640 trials of EEG signals from one participant, and the testing dataset included 320 trials from the same participant. Noise inherent from the power line at frequencies of 50 and 100 Hz was mitigated using a second‐order band‐stop filter. Following noise filtration, the data, formatted as (channels × time steps), underwent random shuffling in sequence prior to the initiation of training. To enhance the robustness of the model, we implemented a data augmentation strategy that involved the incorporation of random noise in 50% of the data samples.

The training deployed the stochastic gradient descent (SGD) optimizer, accommodating a batch size of 32. An initial learning rate of 0.2 was specified, with a cosine annealing learning rate scheduler featuring warm restarts to navigate potential local minima and ensure consistent progression towards optimal convergence. Our model underwent a training duration spanning 500 epochs on an RTX3090, completed within 3 min. Notably, prior to deployment, the model's inference latency was benchmarked at less than 1.5 ms on the same hardware, indicating exceptional readiness for real‐time application.

### 4.8 Immunohistochemistry and Imaging

Six months subsequent to the implantation of the probes, mice underwent perfusion with 1× PBS, followed by 4% paraformaldehyde. Post‐perfusion, brains were extracted and immediately immersed in 4% paraformaldehyde for 48 h to ensure adequate fixation. Afterward, the brains were dehydrated and embedded in wax, followed by cooling at −20°C on a freezing stage until the wax was completely solidified. The brains were then sectioned perpendicularly to the probes using a microtome (RM2016, Leica). The resulting brain slices were then deparaffinized, rehydrated, and subjected to antigen retrieval in sodium citrate buffer (pH 6.0) at a sub‐boiling temperature. After blocking with 3% BSA for 30 min at room temperature, the slices were incubated with the primary antibodies overnight at 4°C. Following this, the slices were washed three times with 1× PBS and incubated with the corresponding secondary antibodies for 50 min at room temperature in darkness. After three 1× PBS washes, the slices were incubated with DAPI solution for 10 min at room temperature in the dark. Fluorescent imaging was performed using a digital slide scanner (Pannoramic MIDI, 3DHISTECH).

## Author Contributions

**Yifei Ye**: data curation, investigation, writing – original draft. **Ye Tian**: investigation, methodology. **Haifeng Liu**: methodology, software, validation. **Jiaxuan Liu**: methodology, software. **Cunkai Zhou**: methodology. **Chengjian Xu**: software. **Ting Zhou**: software. **Yanyan Nie**: resources. **Yu Wu**: methodology. **Lunming Qin**: software. **Zhitao Zhou**: formal analysis, funding acquisition. **Xiaoling Wei**: funding acquisition, resources. **Jianlong Zhao**: funding acquisition, project administration, supervision. **Zhenyu Wang**: software, validation, writing – review and editing. **Meng Li**: resources, software, supervision. **Tiger H. Tao**: funding acquisition, project administration, supervision. **Liuyang Sun**: resources, supervision, writing – review and editing.

## Conflicts of Interest

Prof. Tiger H. Tao and Zhitao Zhou are the co‐founders of Neuroxess Co. Ltd., which may use our probes to carry out research activities or commercial activities based on our results in the future. All other authors declare no conflicts of interest.

## Supporting information



Supporting Information

## Data Availability

The data that support the findings of this study are available from the corresponding author upon reasonable request.
