# Supplementary material for: High‐Precision, Low‐Threshold Neuromodulation With Ultraflexible Electrode Arrays for Brain‐to‐Brain Interfaces
Source: Exploration (Beijing). 2025 Apr 17;5(4):e70040. doi: 10.1002/EXP.70040 (PMC12380067; doi:10.1002/EXP.70040)
Supplement: Supplementary file 1 — Supporting Information [file EXP2-5-e70040-s001.docx]

Supporting Information

**High-precision, low-threshold neuromodulation with ultraflexible electrode arrays for brain-to-brain interfaces**

Yifei Ye^a,^^‡,*^, Ye Tian^a,b,‡^, Haifeng Liu^c,‡^, Jiaxuan Liu^b,d,‡^, Cunkai Zhou^a^, Chengjian Xu^a^, Ting Zhou^e^, Yanyan Nie^f^, Yu Wu^g^, Lunming Qin^h^, Zhitao Zhou^b,d^, Xiaoling Wei^b,d^, Jianlong Zhao^b,d^, Zhenyu Wang^c,*^, Meng Li^b,d,*^, Tiger H. Tao^a,b,d,i,j,k,l,m,n,*^, Liuyang Sun^a,b,d,*^

*^a^* *2020 X-Lab, Shanghai Institute of Microsystem and Information Technology, Chinese Academy of Sciences, Shanghai 200050, China*

*^b^* *School of Graduate Study, University of Chinese Academy of Sciences, Beijing 100049, China*

*^c^* *Intelligent Communication Lab, Shanghai Advanced Research Institute, Chinese Academy of Sciences, Shanghai 201210, China*

*^d^* *State Key Laboratory of Transducer Technology, Shanghai Institute of Microsystem and Information Technology, Chinese Academy of Sciences, Shanghai 200050, China*

*^e^* *School of Microelectronics, Shanghai University, Shanghai 200444, China*

*^f^* *Shanghai Laboratory Animal Research Center, Shanghai 201203, China*

*^g^ Department of Electrical and Computer Engineering, The Ohio State University, Columbus, OH 43210, USA*

*^h^ College of Electronics and Information Engineering, Shanghai University of Electric Power, Shanghai 201306, China*

*^i^* *Center of Materials Science and Optoelectronics Engineering, University of Chinese Academy of Sciences, Beijing 100049, China*

*^j^* *School of Physical Science and Technology, ShanghaiTech University, Shanghai 201210, China*

*^k^* *Center for Excellence in Brain Science and Intelligence Technology, Chinese Academy of Sciences, Shanghai 200031, China*

*^l^* *Neuroxess Co., Ltd. (Jiangxi), Nanchang, Jiangxi 330029, China*

*^m^* *Guangdong Institute of Intelligence Science and Technology, Hengqin, Zhuhai, Guangdong 519031, China*

*^n^* *Tianqiao and Chrissy Chen Institute for Translational Research, Shanghai 200020, China*

^*^ Corresponding authors:

Yifei Ye: [yeyifei@mail.sim.ac.cn](mailto:yeyifei@mail.sim.ac.cn); Zhenyu Wang: [wangzhenyu@sari.ac.cn](mailto:wangzhenyu@sari.ac.cn); Meng Li: [li.meng@mail.sim.ac.cn](mailto:li.meng@mail.sim.ac.cn); Tiger H. Tao: [tiger@mail.sim.ac.cn](mailto:tiger@mail.sim.ac.cn); Liuyang Sun: [liuyang.sun@mail.sim.ac.cn](mailto:liuyang.sun@mail.sim.ac.cn).

^‡^ These authors contributed equally.


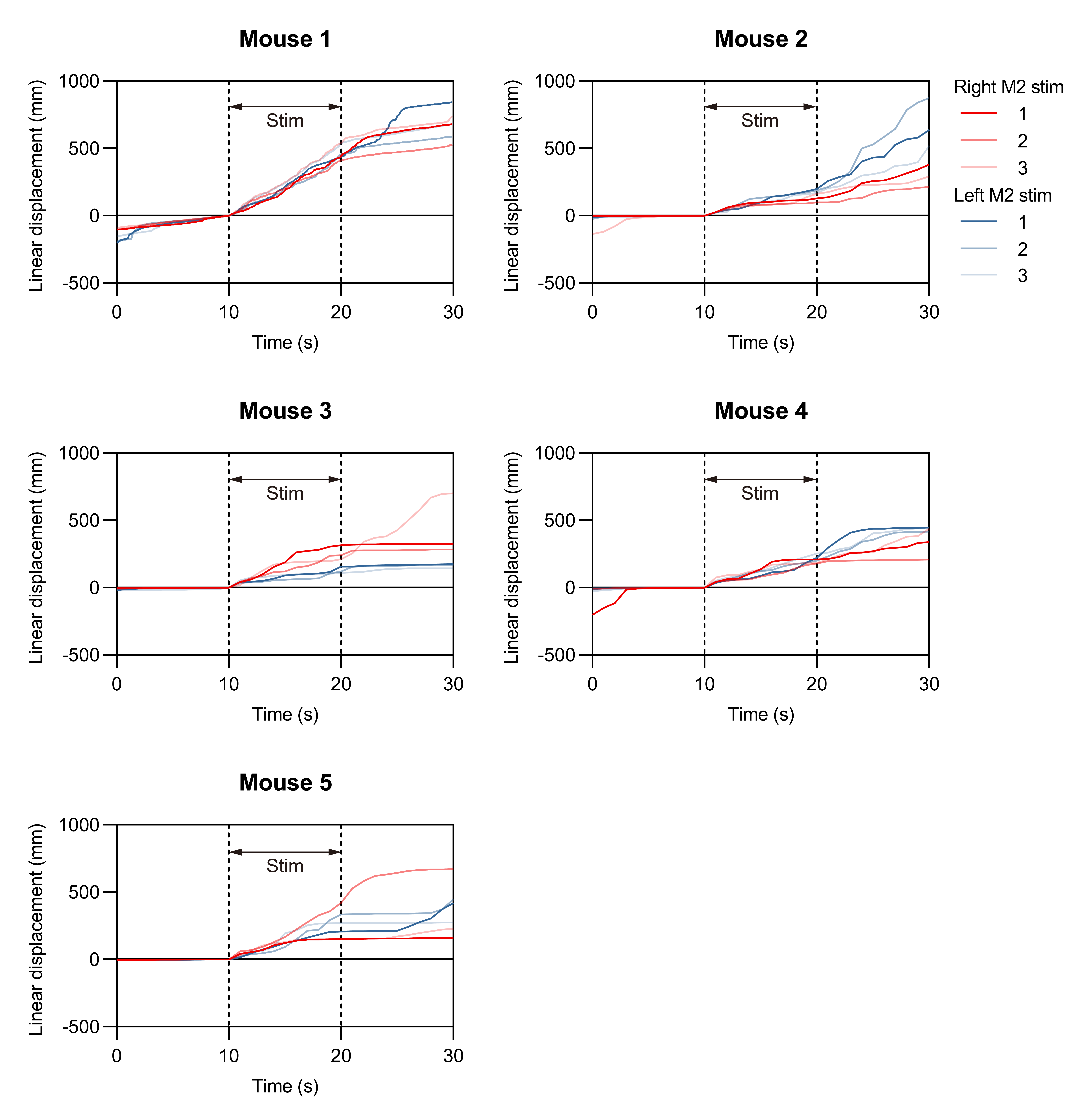


**Figure S1.** Linear displacements of Mice 1-5 before, during, after right or left M2 stimulation.


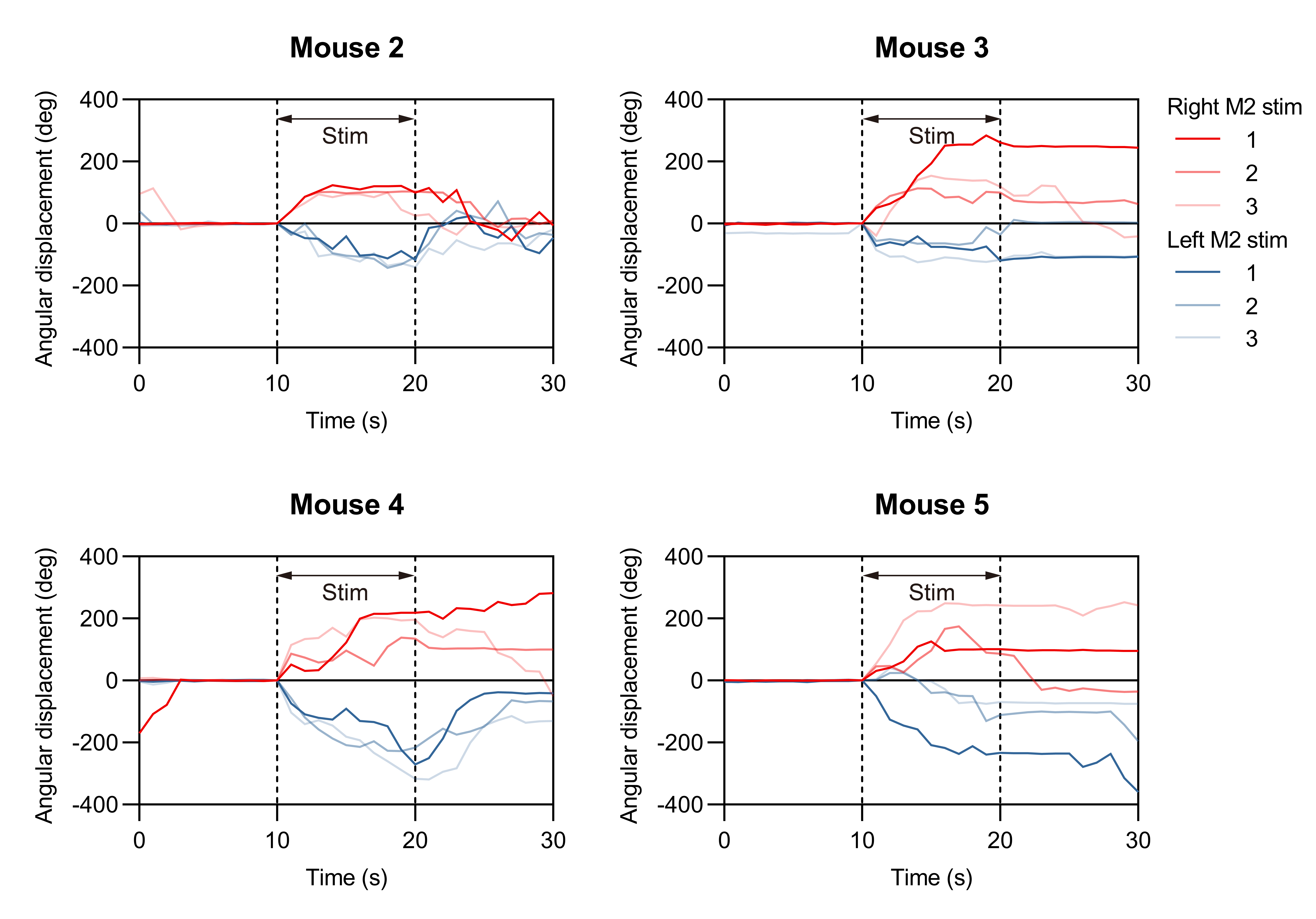


**Figure S2.** Angular displacements of Mice 2-5 before, during, after right or left M2 stimulation.


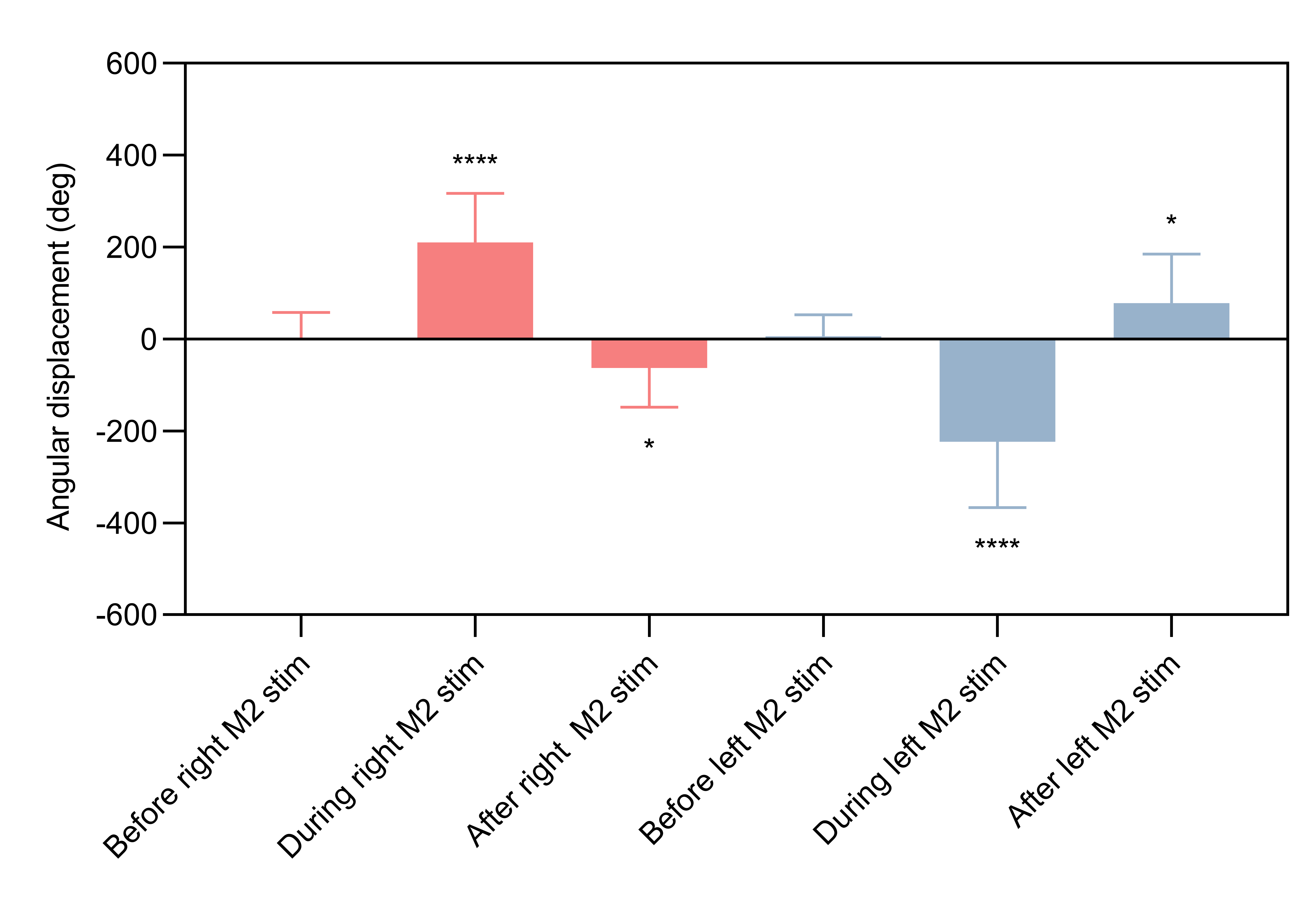


**Figure S3.** Maximum changes in angular displacements before, during, and after right M2 stimulation for five mice. Statistical analysis was performed using two-tailed unpaired Student’s t-tests: ns, p > 0.05; *, p ≤ 0.05; **, p ≤ 0.01; ***, p ≤ 0.001; ****, p ≤ 0.0001.


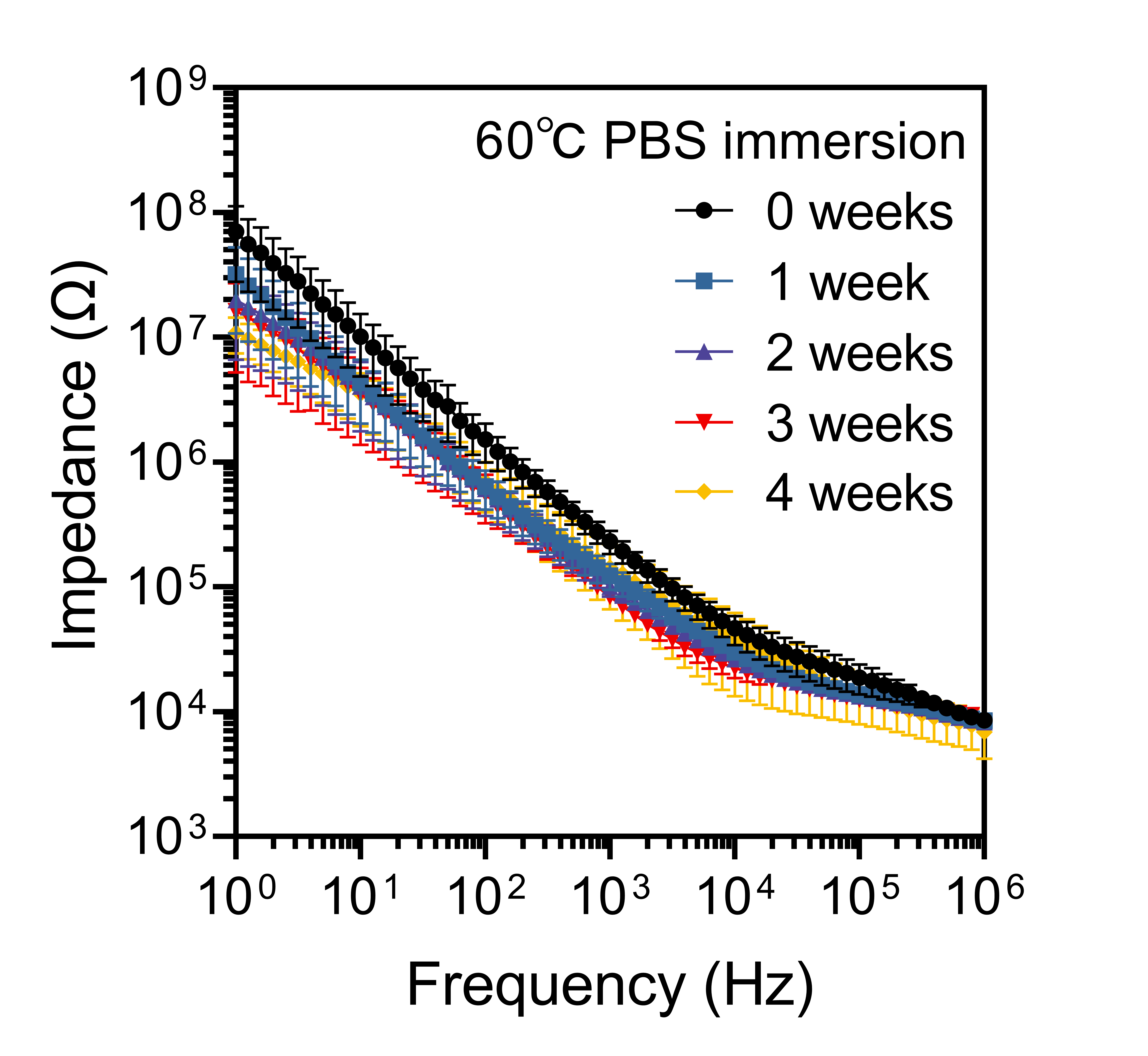


**Figure S4.** Electrical impedance spectroscopy results of PtIr-coated electrodes before and after 1, 2, 3, and 4 weeks of 60°C PBS immersion. Six electrodes were tested.


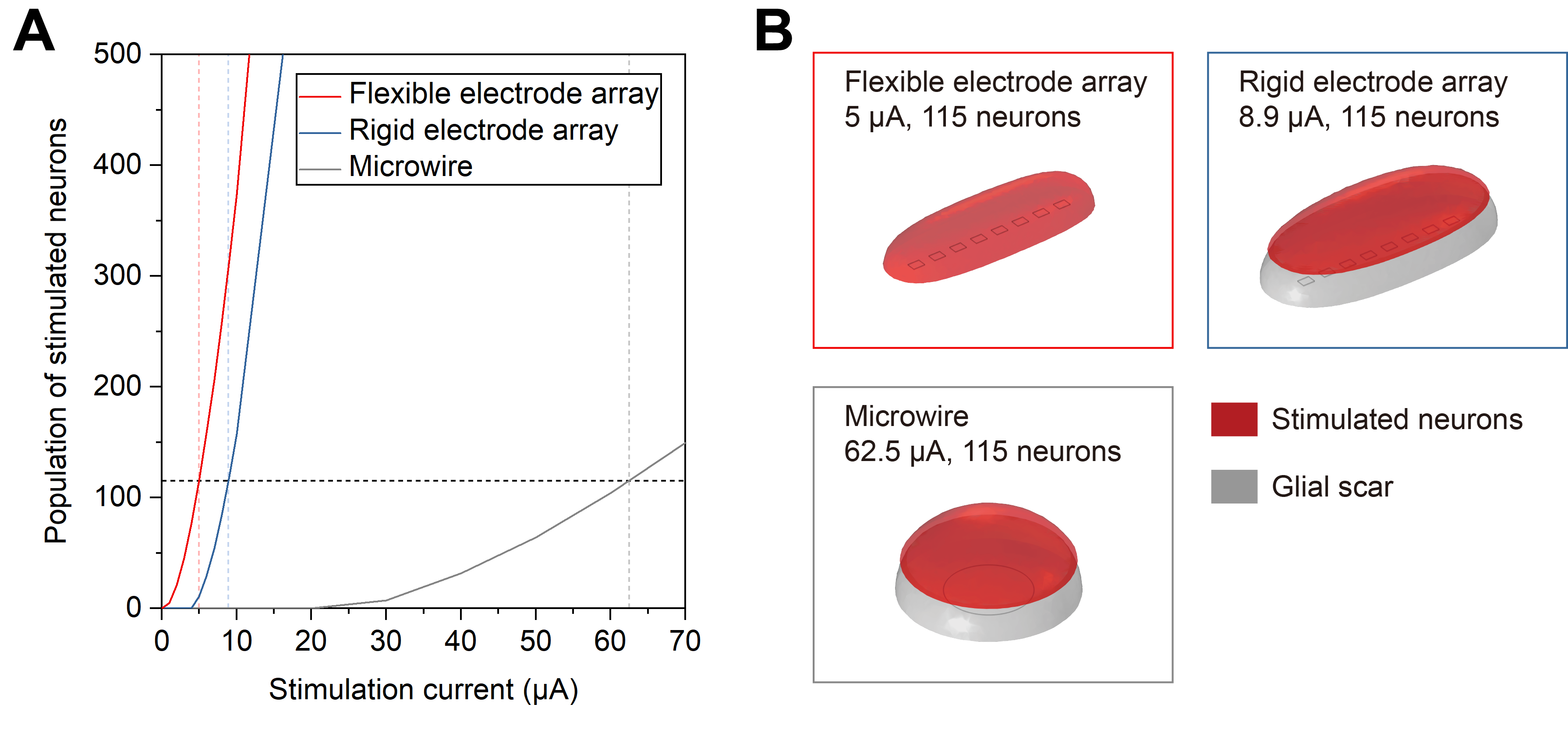


**Figure S5.** Finite element simulation of neuron activation by electrical stimulation using flexible electrode array, rigid electrode array, and microwire. (A) Finite element simulation of the relationship between stimulated neurons and stimulation current using flexible electrode array (8 electrodes in line), rigid electrode array (8 electrodes in line), and a conventional microwire (100 μm diameter). (B) Spatial regions having current densities more than 1000 A m^-2^, where neurons can be stimulated (shown in red), allowing for the stimulation of 115 neurons.


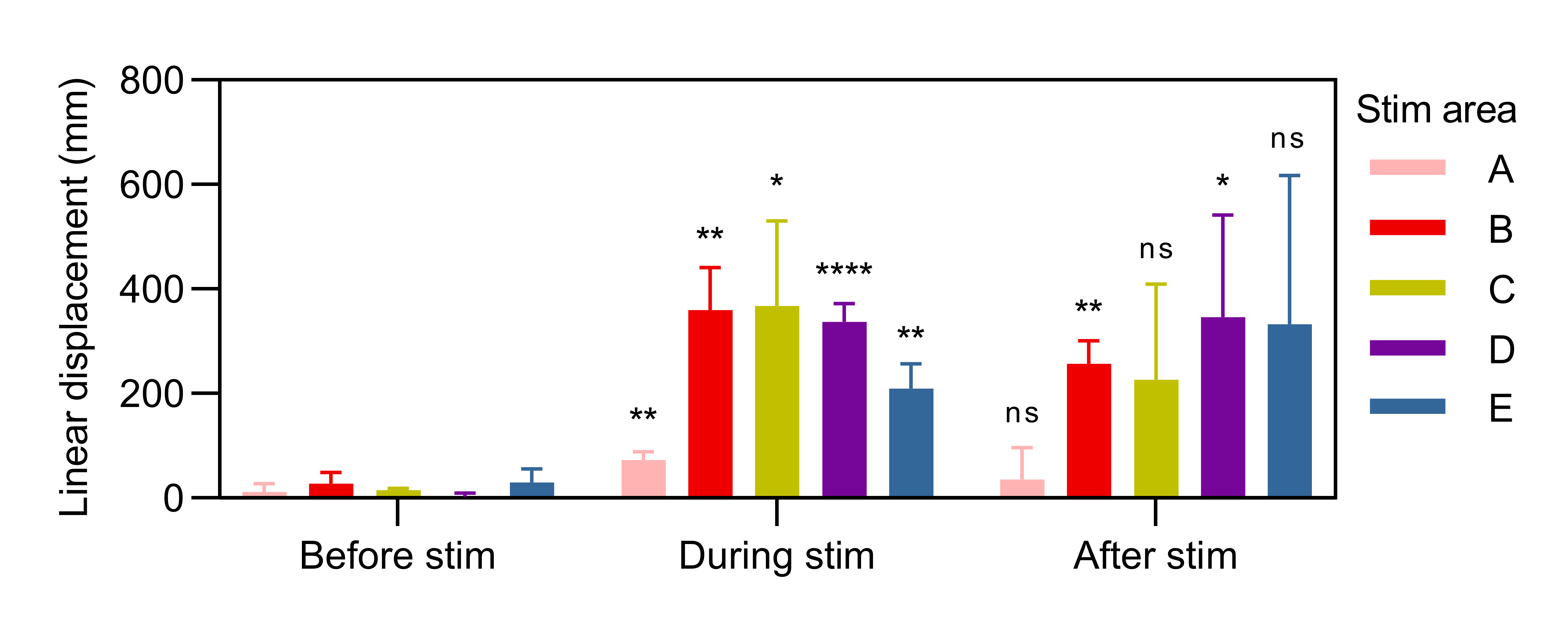


**Figure S6.** Maximum changes in linear displacements before, during, and after stimulation on area A, B C, D, and E. Statistical analysis was performed using unpaired two-tailed Student’s t-test: ns, p > 0.05; *, p ≤ 0.05; **, p ≤ 0.01; ***, p ≤ 0.001; ****, p ≤ 0.0001.


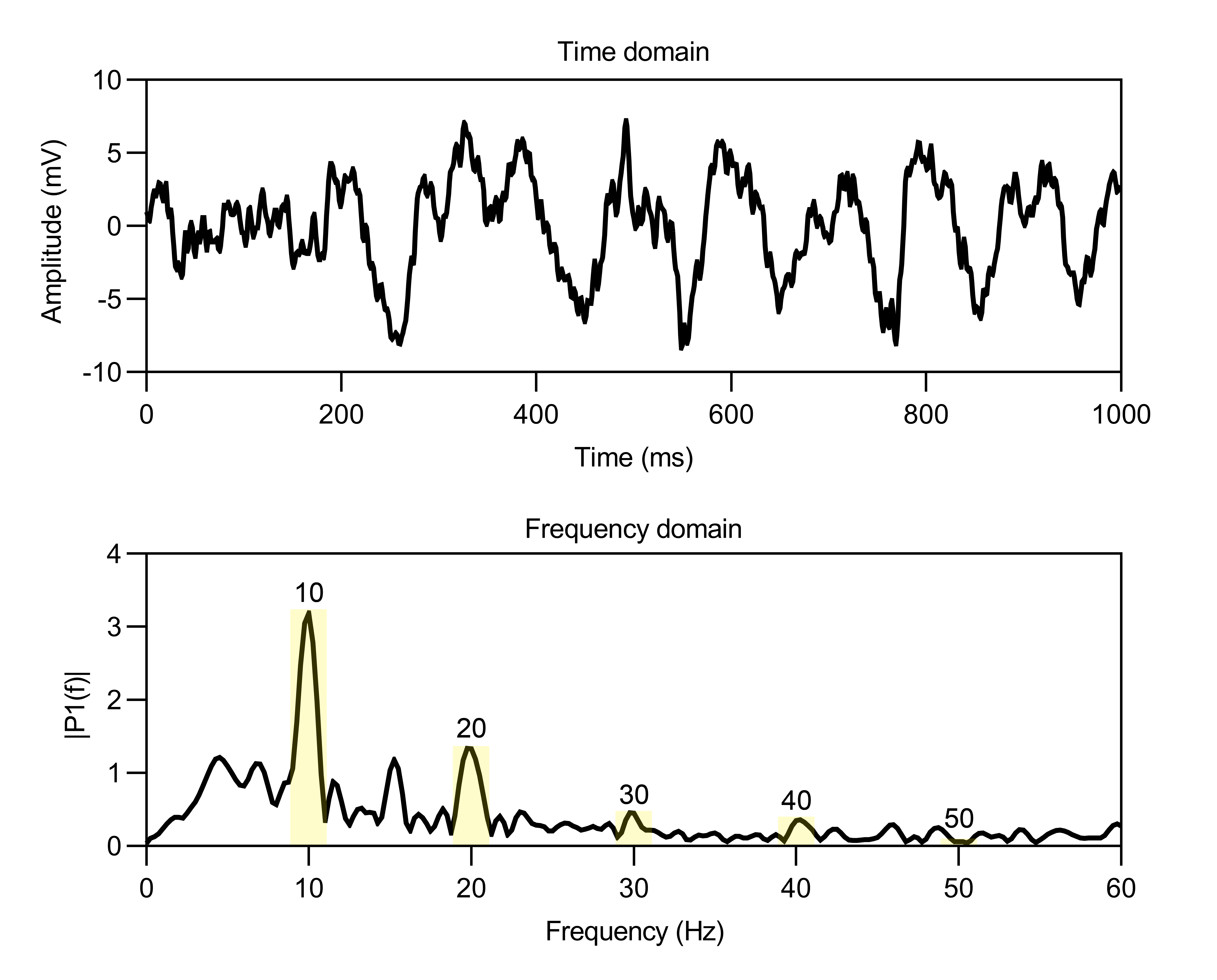


**Figure S7.** A typical SSVEP signal in response to a 10 Hz target represented in time and frequency domains.


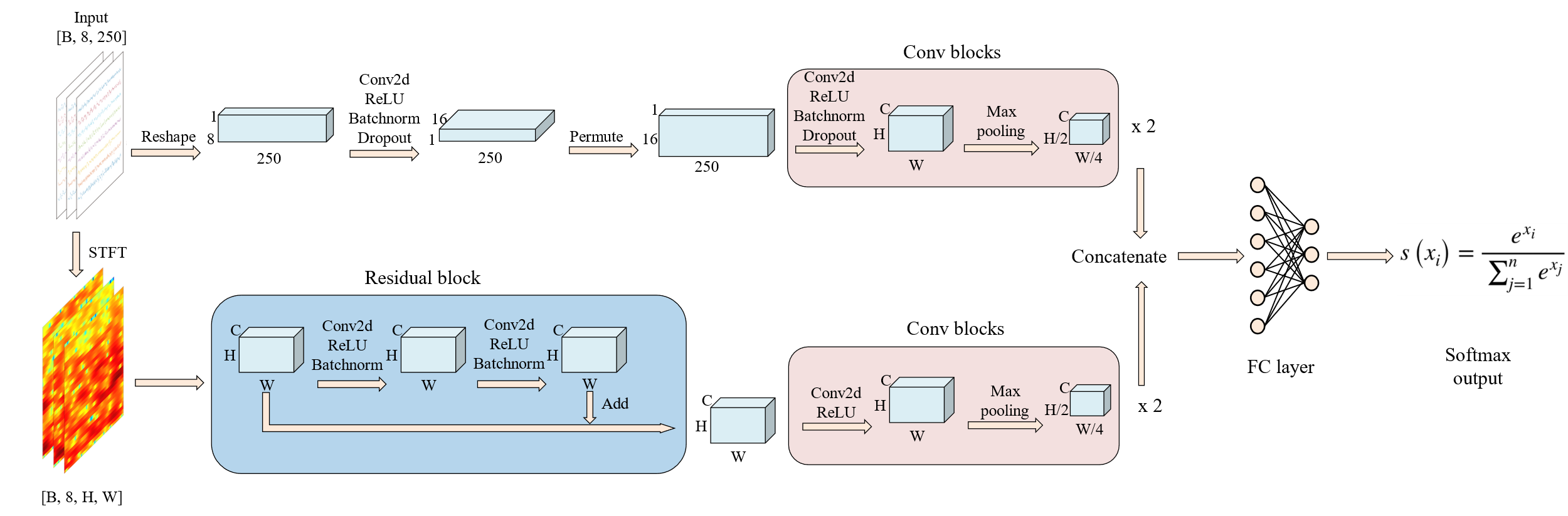


**Figure S8.** Schematic of SSVEP decoding method based on deep neural network.


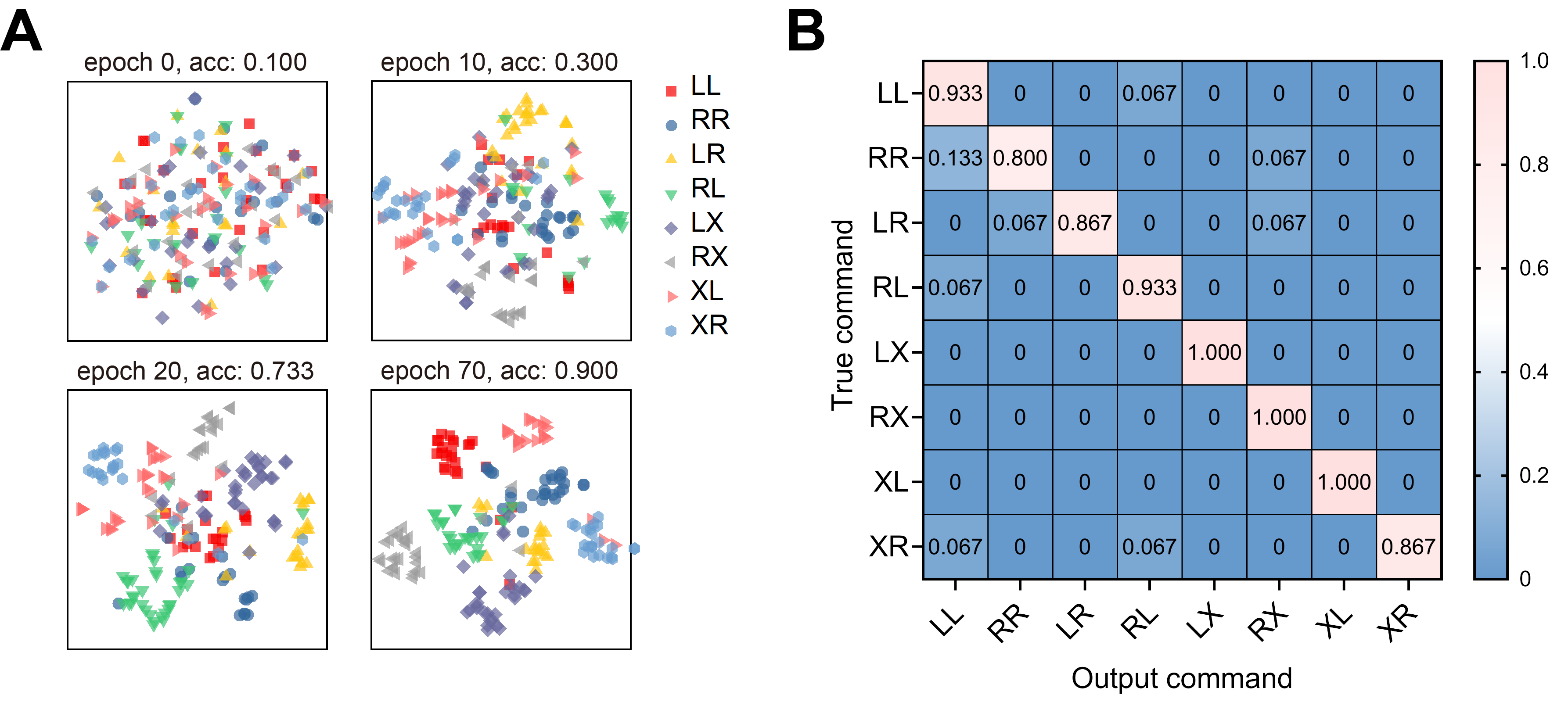


**Figure S9.** Inter-subject validation of SSVEP decoding. (A) The t-distributed Stochastic Neighbor Embedding (t-SNE) visualization with epoch varying from 0 to 70. (B) Averaged confusion matrix between true commands and output commands.


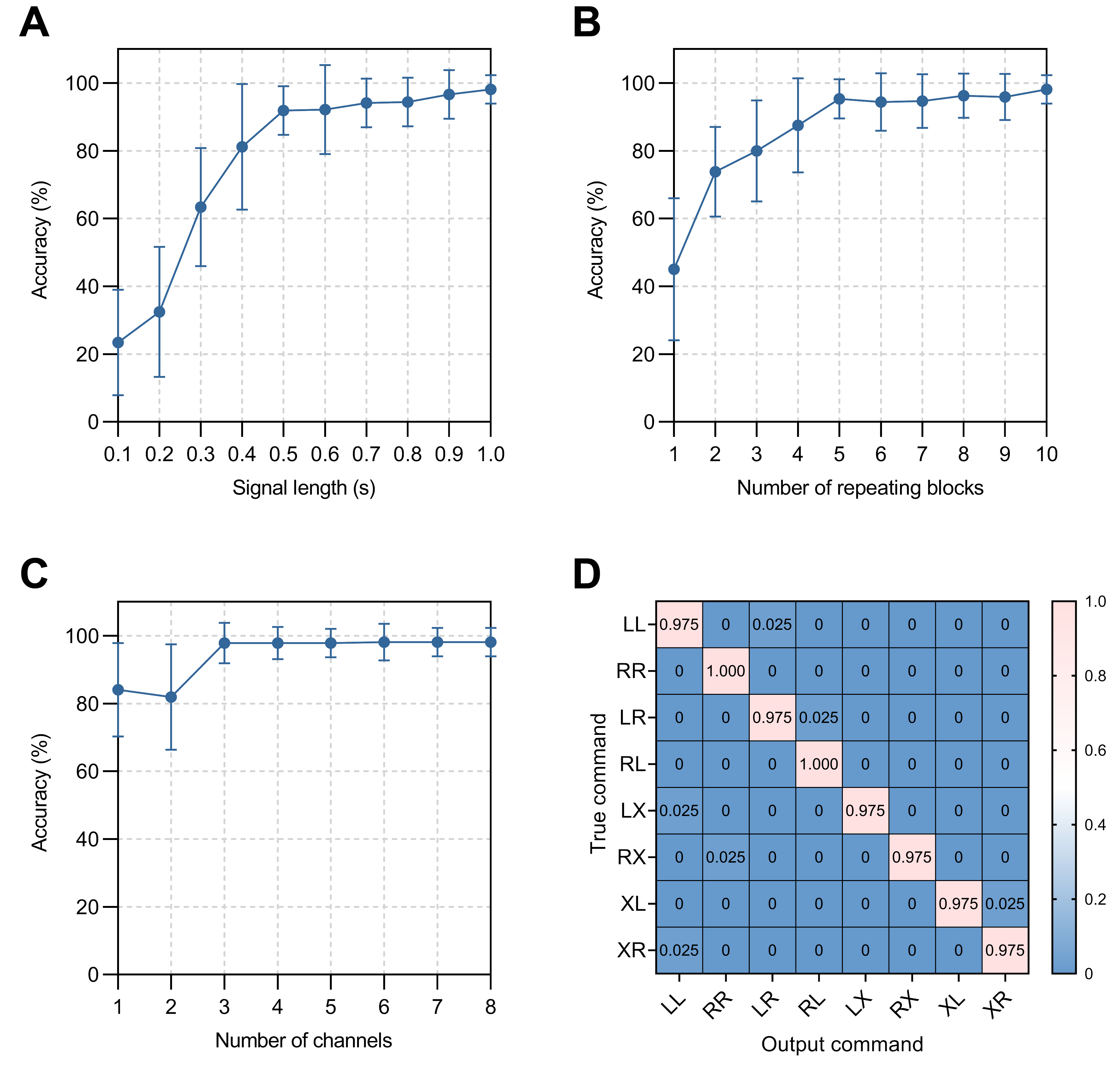


**Figure S10.** Single-subject inter-session validation of SSVEP decoding. (A) Relationship between decoding accuracy and signal length, where the number of channels is 8, the number of repeating blocks is 10. (B) Relationship between decoding accuracy and number of repeating blocks, where the number of channels is 8, the signal length is 1 s. (C) Relationship between decoding accuracy and number of channels, where the signal length is 1 s, the number of repeating blocks is 10. (D) Averaged confusion matrix between true commands and output commands.


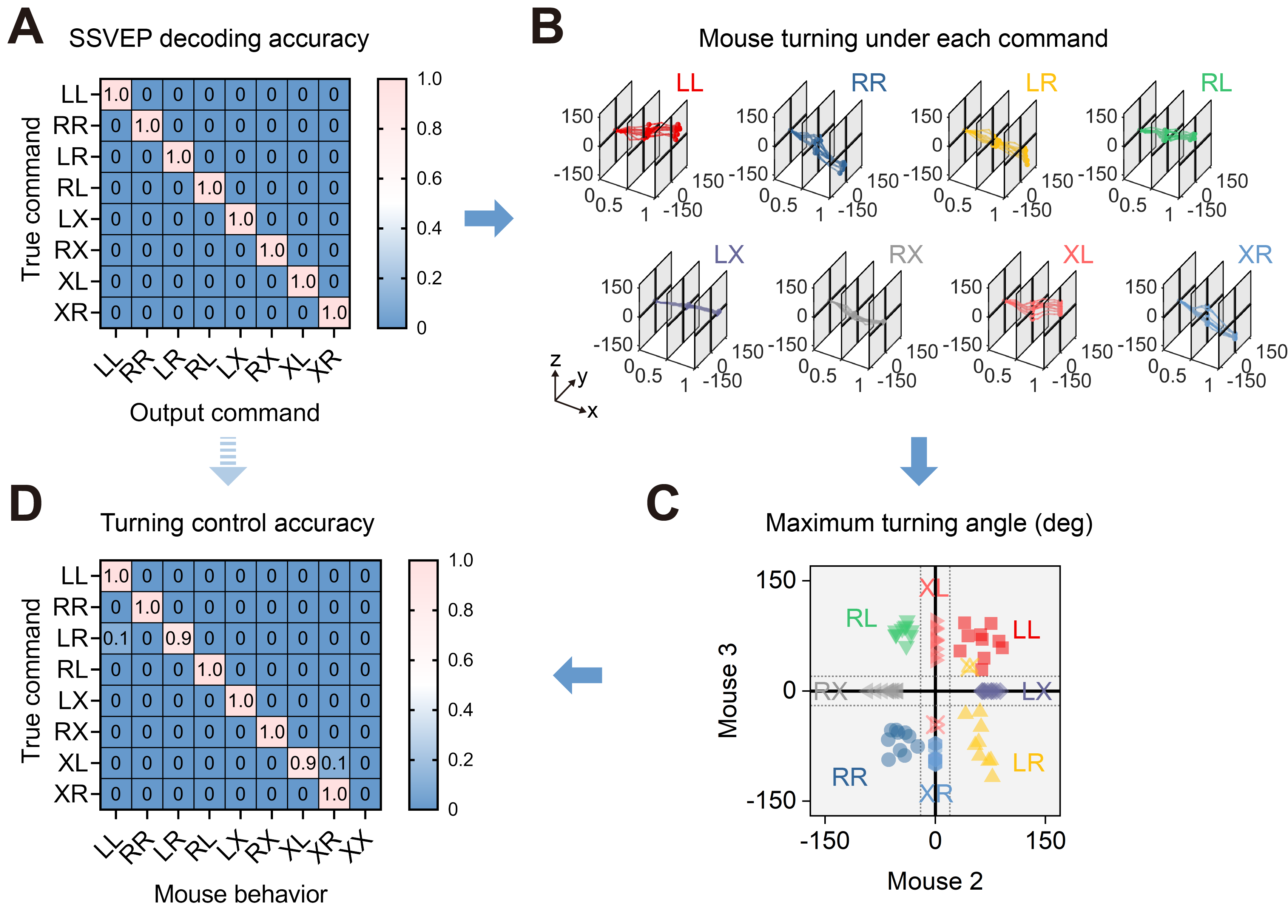


**Figure S11.** Brain-to-brain interface for controlling turning of Mice 2-3 via human brain signals. (A) Confusion matrix of SSVEP decoding accuracy for controlling Mice 2 and 3. (B) Angular displacements of Mice 2 and 3 during 1-second stimulations for each command, where x-axis represents stimulation time (s), y-axis represents angular displacements (deg) of Mouse 2, z-axis represents angular displacements (deg) of Mouse 3. (C) Maximum turning angles achieved by Mice 2 and 3 in response to commands. (D) Confusion matrix of turning control accuracy of B2BI system.


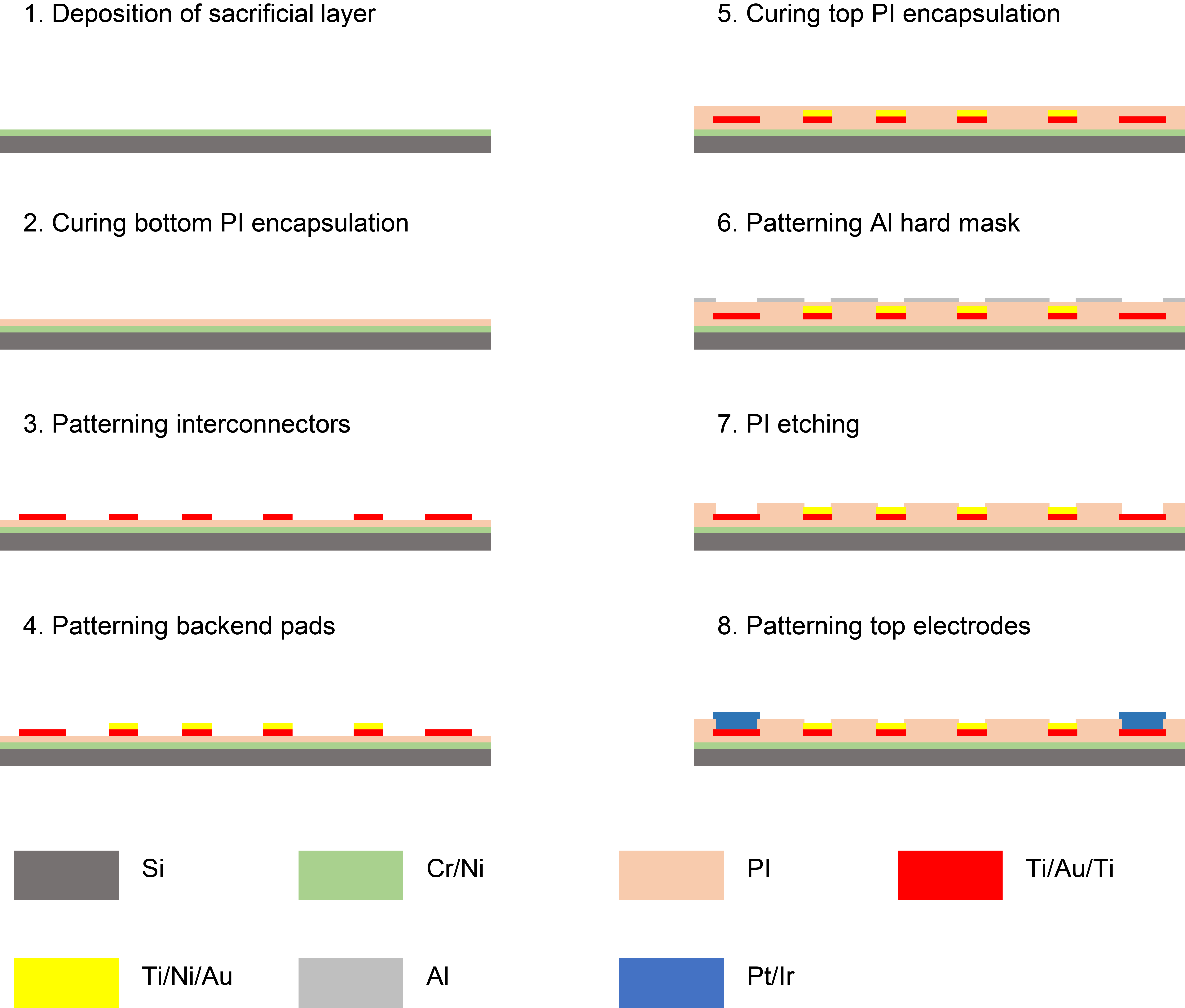


**Figure S12.** Fabrication process of flexible neural probe.
